# A New Eusuchian Crocodyliform with Novel Cranial Integument and Its Significance for the Origin and Evolution of Crocodylia

**DOI:** 10.1371/journal.pone.0030471

**Published:** 2012-01-31

**Authors:** Casey M. Holliday, Nicholas M. Gardner

**Affiliations:** 1 Department of Pathology and Anatomical Sciences, School of Medicine, University of Missouri, Columbia, Missouri, United States of America; 2 Department of Biological Sciences, Marshall University, Huntington, West Virginia, United States of America; Raymond M. Alf Museum of Paleontology, United States of America

## Abstract

Crocodyliforms were one of the most successful groups of Mesozoic tetrapods, radiating into terrestrial, semiaquatic and marine environments, while occupying numerous trophic niches, including carnivorous, insectivorous, herbivorous, and piscivorous species. Among these taxa were the enigmatic, poorly represented flat-headed crocodyliforms from the late Cretaceous of northern Africa. Here we report a new, giant crocodyliform from the early Late Cretaceous (Cenomanian) Kem Kem Formation of Morocco. Represented by a partial braincase, the taxon has an extremely long, flat skull with large jaw and craniocervical muscles. The skull roof is ridged and ornamented with a broad, rough boss surrounded by significant vascular impressions, likely forming an integumentary structure unique among crocodyliforms. Size estimates using endocranial volume indicate the specimen was very large. The taxon possesses robust laterosphenoids with laterally oriented capitate processes and isolated epipterygoids, features allying it with derived eusuchians. Phylogenetic analysis finds the taxon to be a derived eusuchian and sister taxon to *Aegyptosuchus*, a poorly understood, early Late Cretaceous taxon from the Bahariya formation. This clade forms the sister clade of crown-group Crocodylia, making these taxa the earliest eusuchian crocodyliforms known from Africa. These results shift phylogenetic and biogeographical hypotheses on the origin of modern crocodylians towards the circum-Tethyean region and provide important new data on eusuchian morphology and evolution.

## Introduction

Crocodyliforms (Archosauria) achieved extraordinary success during the Cretaceous period, during which they occupied numerous trophic niches on virtually every continent. In particular, early Late Cretaceous northern African sedimentary deposits have revealed a diverse assemblage of aquatic, terrestrial, predatory and herbivorous crocodyliforms [1]–[8]. These crocodyliforms lived alongside numerous other vertebrates, including sarcopterygian fishes, turtles, plesiosaurs, snakes and varanoid lizards, pterosaurs, and sauropod and theropod dinosaurs [9]–[11] within a continental and freshwater deltaic environment [12].

Perhaps the most captivating of this fauna were the stomatosuchids, a poorly represented clade of giant, flat-snouted crocodyliforms. First described by Stromer [1], [13], *Stomatosuchus inermis* from the Bahariya Formation of Egypt was unique among crocodyliforms in having an extraordinarily long, broad, flat skull and thin mandibular rami. The remainder of the skull included a crushed palate and rostrum, and only a fragmentary braincase housing the eustachian opening, occipital condyle and partial skull roof. Sadly, this type material, along with other invaluable fossils, was destroyed during the Allied bombings of Munich in 1944. Recently, Sereno and Larsson [8] described putative stomatosuchid mandibular material from the Cenomanian of Morocco and Niger (*Laganosuchus thaumastos* and *L. maghrebensis*) consisting of well- preserved, long, thin mandibular rami with small conical teeth, and a slight, U-shaped mandibular symphysis, thus shedding new light on this poorly represented group of crocodyliforms.

Surviving the destruction of the museum in 1944 was the partial skull roof of a second unusual crocodyliform, *Aegyptosuchus peyeri* (BSPG [Bayerische Staatssammlung für Paläontologie und Geologie] 1912 VIII 177) [12]. The specimen has a characteristically dorsoventrally thick skull table and small, constricted dorsotemporal fossae, similar to that described for *Stomatosuchus* [1]. Additional material (though also destroyed) originally referred to *Aegyptosuchus* included an ectopterygoid, articular, teeth and isolated cervical, presacral, sacral, and caudal vertebrae [12]. The strong procoelus vertebrae suggested *Aegyptosuchus* may have been a derived neosuchian crocodyliform [13]. However, since the loss of these important fossils, informative additional material from the cranium of *Stomatosuchus* or *Aegyptosuchus* has been lacking, understandably hampering phylogenetic and ecological insights into these taxa.

**Figure 1:**
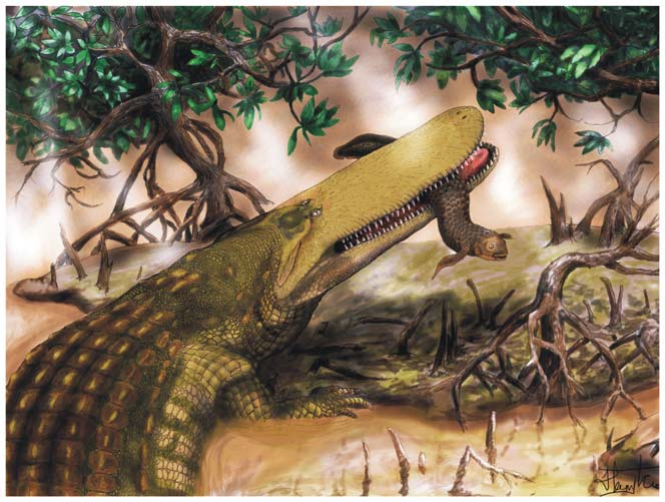
Life restoration of *Aegisuchus witmeri*, a giant, flat-headed, ornamented crocodyliform from the Late Cretaceous of northern Africa. Original artwork by Henry P. Tsai, University of Missouri.

**Figure 2:**
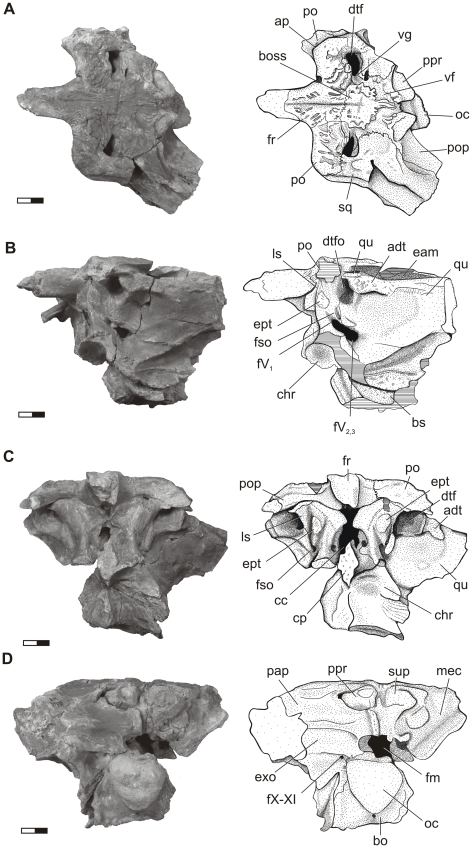
Cranial anatomy of *Aegisuchus witmeri*. **Left**, photograph, **Right**, interpretive illustration. **A**, dorsal view; **B**, left lateral view; **C**, rostral view; **D**, caudal view. Scale bar equals 1 cm. **Abbreviations**: **adt**, adductor tubercle; **bo**, basioccipital; **boss**, integumentary boss; **bs**, basisphenoid; **cc**, cranial cavity; **chr**, choanal recess; **cp**, cultriform process; **dtf** dorsotemporal fenestra; **dtfo**, dorsotemporal fossa; **eam**, external auditory meatus; **ept**, epipterygoid; **fm**, foramen magnum; **fr**, frontal; **fso**, supraorbital nerve foramen; **fX-XI**, foramen for cranial nerves X and XI; **fV_2,3_**, maxillomandibular foramen; **gV_1_**, ophthalmic groove; **ls**, laterosphenoid; **mec**, middle ear cavity; **oc**, occipital condyle; **pap**, paroccipital process; **po**, postorbital; pop, postorbital process; **ppr**, post-occipital protuberance; **qu**, quadrate; **sq**, squamosal; **sup**, supraoccipital; **vf**, vascular fossa; **vg**, vascular groove.

**Figure 3:**
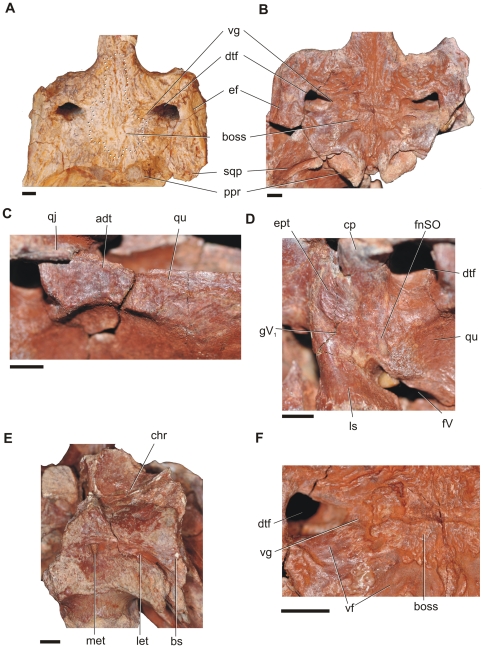
Osteological correlates in *Aegisuchus witmeri*. **A**, dorsal view of *Aegyptosuchus peyeri* illustrating less-distinct cranial boss. **B**, dorsal view of *Aegisuchus witmeri* with distinct integumentary boss surrounded by vascular fossa. **C**, left lateral view of hypertrophied jaw muscle scar and adductor tubercle on the rostrolateral surface of the quadrate. **D**, left lateral view of isolated epipterygoid and trigeminal foramen. **E**, left, lateral view of temporal region with passages for important neuromuscular structures with insets for C, D. **F**, dorsal view highlighting reconstructed vasculature and musculature of the skull roof and occipital region. **Abbreviations**: **adt**, adductor tubercle; **boss**, integumentary boss; **bs**, basisphenoid; **chr**, choanal recess; **cp**, capitate process; **dtf** dorsotemporal fenestra; **eam**, external acoustic meatus; **ef**, fossa for external ear flap; **ept**, epipterygoid; **fV**, trigeminal foramen; **fnSO**, supraorbital nerve foramen; **gV_1_**, ophthalmic groove; **let**, lateral Eustachian tube; **ls**, laterosphenoid; **met**, median Eustachian tube; **ppr**, postoccipital protuberance; **qj**, quadratojugal; **qu**, quadrate; **sqp**, squamosal prong; **vf**, vascular fossa; **vg**, vascular groove.

**Figure 4:**
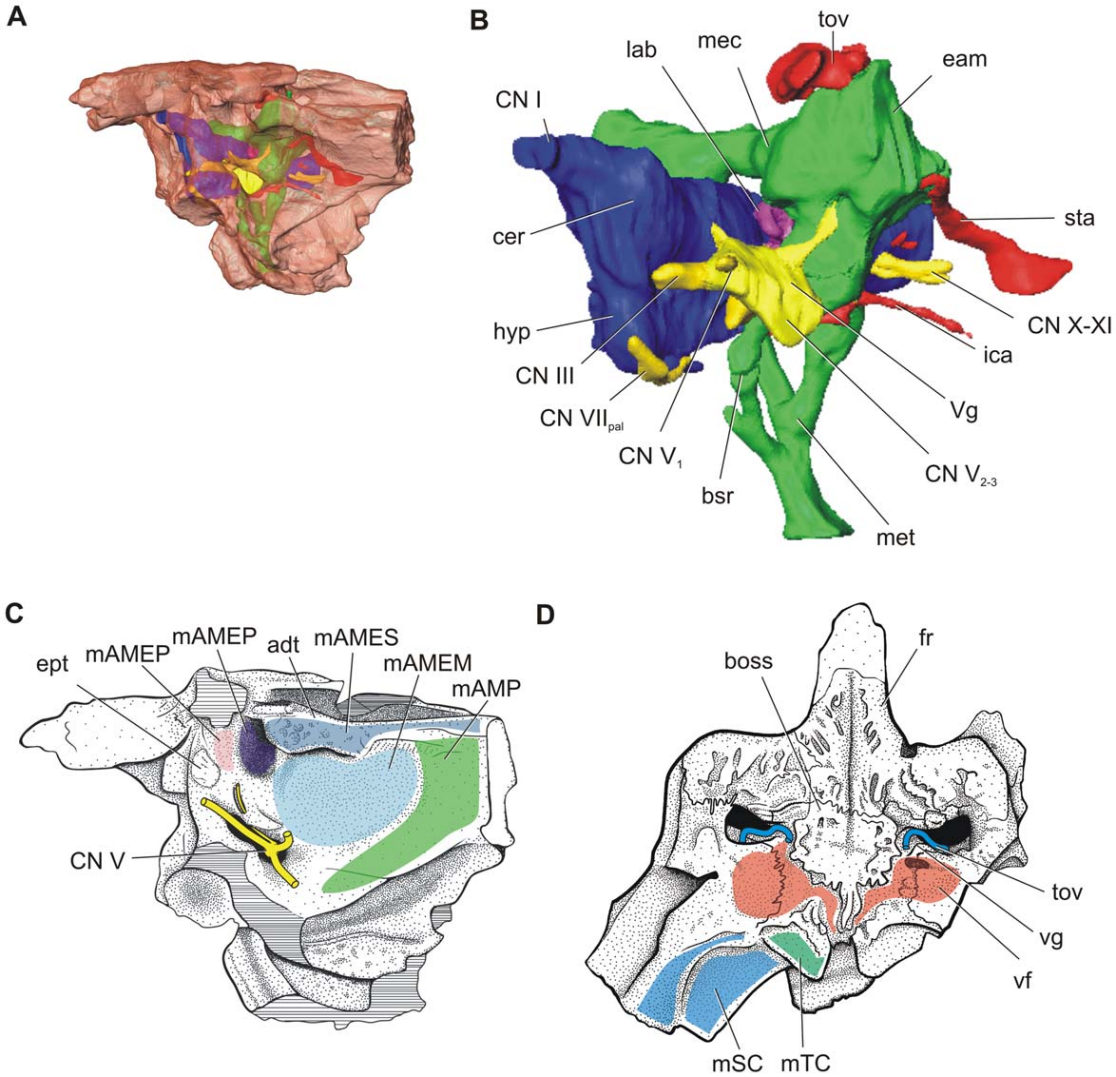
3D reconstruction of *Aegisuchus witmeri* with brain endocast and other soft tissue structures in left, oblique lateral view. Brain volume (∼40 cm^3^) was used to estimate head length. **H**, scout image of G. **Abbreviations**: **adt**, adductor tubercle; **boss**, integumentary boss; **bsr**, basisphenoid recess; **cer**, cerebrum; **CN I**, olfactory tract; **CN III**, oculomotor nerve; **CN V_1_**, ophthalmic nerve; **CN V_2–3_**, maxillary nerve; **CN V_3_**, mandibular nerve; **CN VII_pal_**, palatine ramus of facial nerve; **CN X–XI**, vagus and accessory nerves; **dtf** dorsotemporal fenestra; **eam**, external acoustic meatus; **ept**, epipterygoid; **fV**, trigeminal foramen; **fr**, frontal; **fnSO**, supraorbital nerve foramen; **hyp**, hypophysis (pituitary gland); **ica**, internal carotid artery; **lab**, labyrinth; **mAMES**, m. adductor mandibulae externus superficialis; **mAMEM**, m. adductor mandibulae externus medialis; **mAMEP**, m. adductor mandibulae externus profundus; **mAMP**, m. adductor mandibulae posterior; **mSC**, m. splenius capitis; **mTC**, m. transverses capitis; **mec**, middle ear cavity; **met**, median eustachian tube; **mPSTs**, m. pseudotemporalis superficilais; **sta**, stapedial artery; **sq**, squamosal; **tov**, temporoorbital vessels; **Vg**, trigeminal ganglion; **vf**, vascular fossa; **vg**, vascular groove.

**Figure 5:**
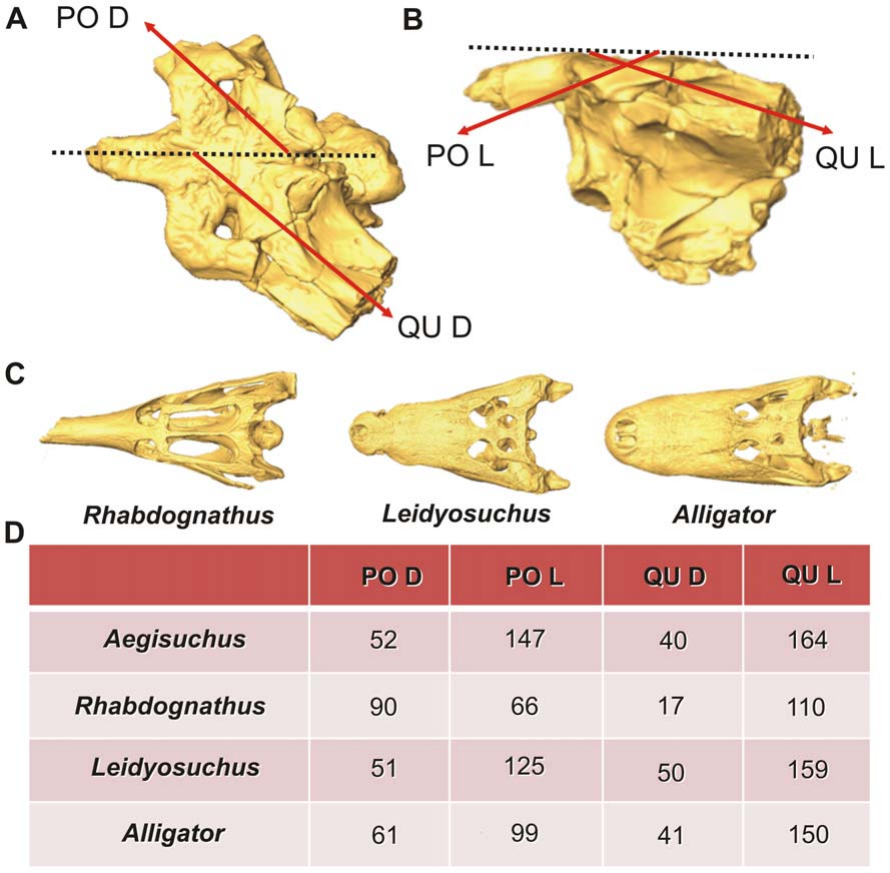
Angles of the postorbital and quadrate/paroccipital processes of *Aegisuchus witmeri* and other representative crocodyliforms illustrating the flatness of the skull of *A. witmeri*. **A**, Dorsal view, angles of postorbital (PO D) and quadrate (QU D). **B**, Lateral view, angles of postorbital (PO L) and quadrate (QU L). **C**, the dyrosaur (Neosuchia) cf. *Rhabdognathus* sp. (CNRST-SUNY-190), the basal brevirostrine crocodylian *Leidyosuchus canadensis* (ROM 1903), and the extant crocodylian *Alligator mississippiensis* (Holliday Lab AL022). **D**, Table with angle values of each measurement in representative crocodyliform taxa.

**Figure 6:**
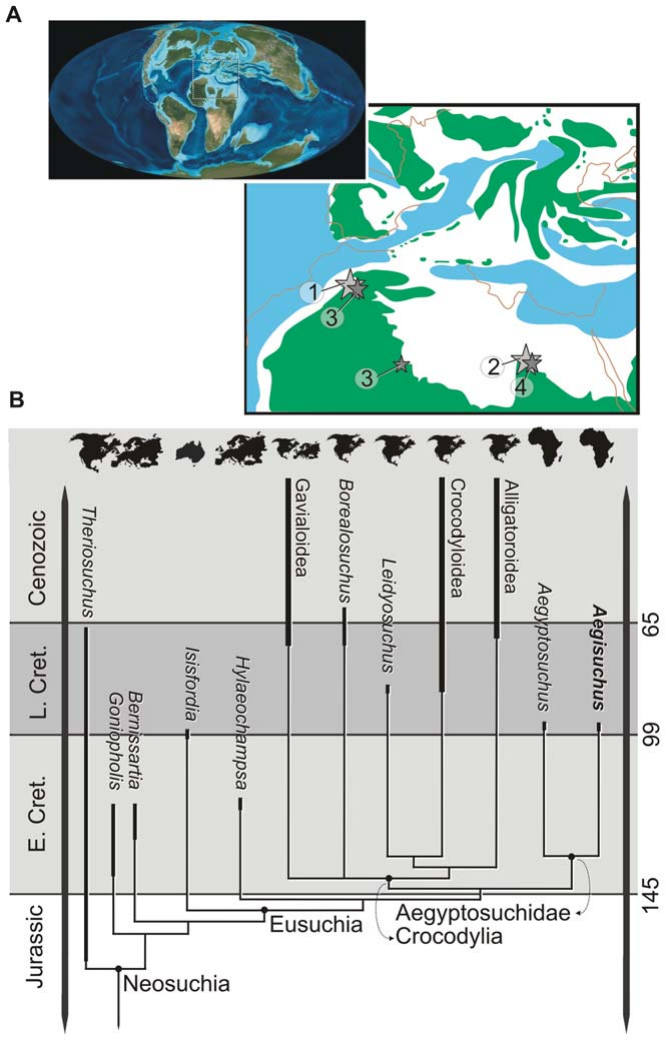
Biogeographic and phylogenetic relationships of eusuchian crocodyliform *Aegisuchus witmeri* and related taxa. **A**, Early Late Cretaceous Mollweide projections [41] of circum-Tethyean continental geography with discoveries of aegyptosuchid and stomatosuchid taxa. **1**, *Aegisuchus witmeri*; **2**, *Aegyptosuchus peyeri*; **3**, *Laganosuchus thaumastos*; **4**, *Stomatosuchus inermis*. **B**, Biogeographical provinces, phylogenetic, relationships, and stratigraphic ages of *Aegisuchus* and relevant crocodyliforms. Stratigraphic data from [38]–[41]. See Figs 7, 8, 9 for complete results of phylogenetic analysis.

**Figure 7:**
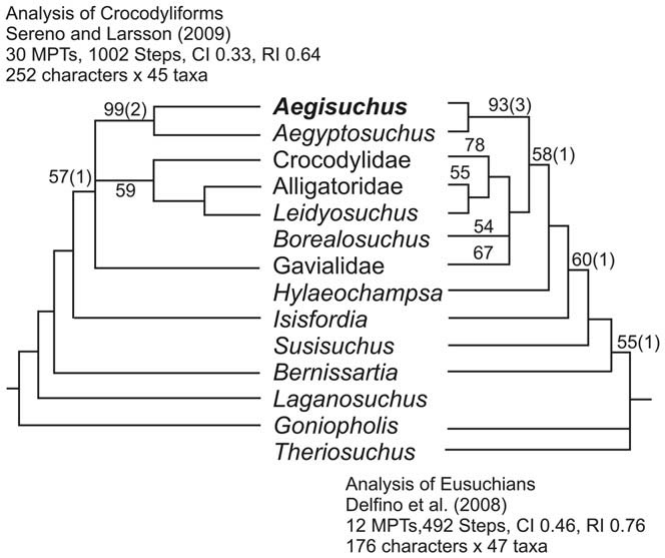
Evolutionary relationships and details of phylogenetic analysis of *Aegisuchus witmeri* using two different analyses. **Left**, analysis of relationships of *A. witmeri* within Crocodyliformes using matrix from Sereno and Larsson [8]. **Right**, analysis of relationships of *A. witmeri* within Eusuchia using matrix from Delfino et al. [18]. These trees have been cropped to include only taxa near the neosuchian/eusuchian transition. Includes step lengths (Bremer decay indices).

**Figure 8:**
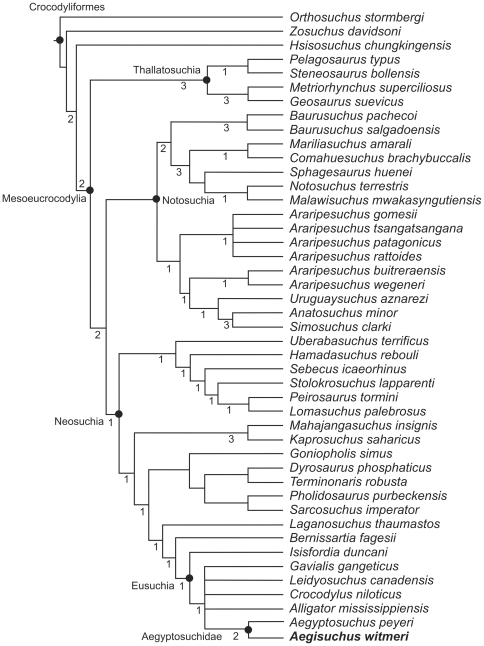
Complete phylogenetic analysis of *Aegisuchus witmeri* and Crocodyliforms using Sereno and Larsson [**8**] with Bremer decay indices.

**Figure 9:**
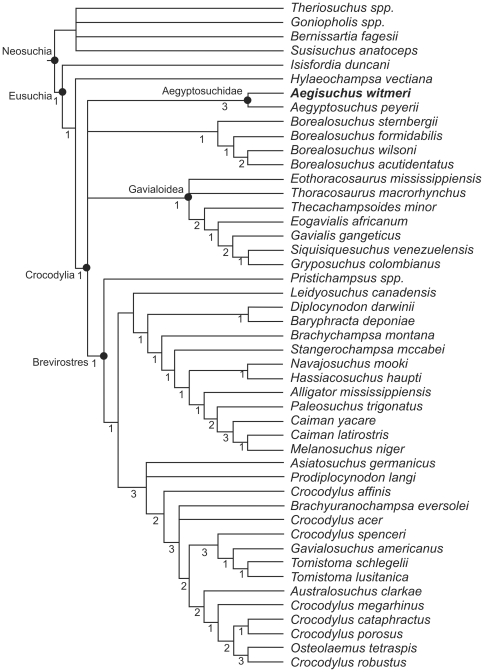
Complete phylogenetic analysis of *Aegisuchus witmeri* and Eusuchia using Delfino et al. [**18**] with Bremer decay indices.

Here we report on a new species of crocodyliform (Figs. 1, 2, 3, 4, 5) from the Cenomanian (98–93 Ma) Kem Kem Formation of southeastern Morocco (Fig. 6) which shares significant features with *Aegyptosuchus* and the described *Stomatosuchus* material. Phylogenetic analysis (Figs. 7, 8, 9) places *Aegisuchus witmeri* gen. et sp. nov. as a member of the Aegyptosuchidae, here considered as the eusuchian sister clade to Crocodylia, but fails to recover Aegyptosuchidae and the putative stomatosuchid *Laganosuchus* as a clade. *Aegisuchus witmeri* is characterized by an especially flat skull, adaptations for strong jaw opening, and what appears to be a cranial, vascular integumentary structure novel to crocodyliforms (Figs. 2, 3, 4). Until this discovery, the immediate relatives of crown-group crocodylians have been unknown from African Cretaceous sediments, suggesting that modern crocodylian origins were limited to Laurasia [6], [8], [13]–[18]. The new species described here challenges this biogeographical hypothesis but also reveals that crocodyliforms may have evolved display structures only previously seen in their panavian relatives.

### Systematic Paleontology

Archosauria Cope, 1869 (sensu Gauthier and Padian, 1985)

Crocodyliformes Hay, 1930 (sensu Benton and Clark, 1988)

Mesoeucrocodylia Whetstone and Whybrow, 1983 (sensu Sereno et al., 2001)

Eusuchia Huxley, 1875 (sensu Brochu, 2003)

Aegyptosuchidae Kuhn, 1936 *Aegisuchus witmeri* gen.et sp. nov.

New Genus ZooBank LSID:

urn:lsid:zoobank.org:act:D57A835B-26D1-42A5-9DAC-C217B4EB2CCB

New species ZooBank LSID:

urn:lsid:zoobank.org:act:2B10FEDB-DF73-4579-BB8A-005B527D44D5

#### Etymology

*Aegis*, shield (Greek), describing the integumentary boss on the skull roof; *souchus*, crocodile (Greek); *witmeri*, in honor of Lawrence M. Witmer, whose mentorship and contributions to archosaur cranial anatomy enabled the identification and interpretation of this specimen.

#### Holotype

ROM 54530 (Royal Ontario Museum, Toronto, Canada), partial braincase of a large individual with skull roof, temporal, and occipital regions.

#### Locality

The specimen is from the early Late Cretaceous (Cenomanian) Kem Kem Formation of southeastern Morocco, a continental and deltaic environment [16]. The specimen was collected by commercial collectors and obtained by curators from the Royal Ontario Museum.

#### Diagnosis

*Aegisuchus* possesses the following autapomorphies: raised, rugose boss with surrounding smooth fossa on dorsal surface of parietal; large, quadrangular adductor tubercle on rostrolateroventral surface of quadrate; ovate, isolated epipterygoid on rostrolateral surface of laterosphenoid; rounded torus on rostrolateral edge of dorsotemporal fenestra.

## Results

### Description and Comparisons

*Aegisuchus* is represented by a single mostly-complete braincase from a large individual. The skull tables in both *Aegisuchus* and *Aegyptosuchus peyeri*, a related, coeval species from the Cenomanian Bahariya Formation of Egypt [12], are markedly dorsoventrally thick and buttressed by robust laterosphenoids and are adorned with rugose bosses on the parietals. Both species have enlarged exoccipital protuberances, an expanded, dorsally facing occipital region for large epaxial muscles, and isolated epipterygoids [19]. Derived features shared between the two aegyptosuchid taxa include a postorbital-squamosal suture which passes medially to skull table, and a laterally-oriented laterosphenoid capitate process, a feature convergent upon that present in gavialoids.

The frontals of *Aegisuchus* are sculpted by a series of thick obliquely-oriented parallel ridges that emanate from a deep, midline trough and then expand caudally onto the parietals (Figs. 2, 3, 4). The dorsal surfaces of the parietals are covered by a large, circular, rugose boss that merges rostrally with the frontal ridges and tapers caudally, abruptly ending as a midline finger-like projection near the occiput. The boss is rimmed by small lobate processes which protrude into a broad, shallow fossa that circumscribes the boss and the remainder of the skull roof. The fossa communicates with deep vascular grooves of the temporoorbital vessels that emerge from small, constricted, vertical-walled, dorsotemporal fossae (Fig. 5). The surface texture of the boss suggests that a thickened layer of tightly adherent integument, similar to that seen in other surfaces of crocodyliform skulls whereas the surrounding vasculature suggests a more complicated integumentary structure was present. The skull roof of *Aegyptosuchus* is also similarly textured to the condition seen in *Aegisuchus*; however the central boss is not as well demarcated and lacks a clear, circumscribing vascular fossa. Stromer described *Stomatosuchus* as having a weakly-grooved frontoparietal region, textured skull roof, and widely spaced, small, mediolaterally-ovate dorsotemporal fossae [1], [23], features shared with *Aegisuchus* and *Aegyptosuchus*, indicating that despite poor current phylogenetic resolution uniting the three taxa, they are likely closely related.

In caudal view, the supraoccipital is triangular in shape, and both *Aegisuchus* and *Aegyptosuchus* have two prominent post-occipital protuberances. The supraoccipital is not exposed on the skull roof. The lateral corners of the element bear deep pits for the passage of the occipital veins. The left exoccipital is well preserved and extends medially to its vertical suture with the missing contralateral exoccipital; these elements formed the dorsal and lateral margins of the foramen magnum. A prominent, short crest parallels the midline suture dorsal to the foramen magnum, and a small fossa and crest lateral to the foramen outline the ligamentous attachments of the neural arches of the atlas. The exoccipital extends laterally, forming the caudolaterally projecting paroccipital process. The process is robust, square in cross-section, and is shallowly swept caudolaterally. A prominent, mediolaterally oriented ridge divides the paroccipital process into a dorsal, dorsally-facing surface and a ventral, caudally-facing surface. The ventral half has a deep, mediolaterally long rectangular fossa, bounded ventrally by the foramen for cranial nerves IX, X, XI and the jugular vein and more medially by the foramen for XII, all of which are nestled in a shallow fossa near the suture with the basioccipital. Lateral to these foramina is the foramen for the internal carotid artery, which enters the skull within a fossa under an overlying rim of the paroccipital process.

The basioccipital is large and trapezoidal in caudal profile. The occipital condyle is very large and bulbous, although the articular surface is mostly destroyed. Ventrally, the basioccipital extends rostrally to surround a large median eustachian opening. Lateral to the opening are the two wide and deep lateral eustachian grooves which pass within the basioccipital, caudal to the suture for the basisphenoid and rostral to the two large lateral basioccipital protuberances. The basisphenoid is rostrocaudally long and broad. Only the dorsal portion of the element remains, revealing a large choanal recess. The caudolateral flanges of the basisphenoid extend between the basioccipital and the caudomedial wing of the pterygoid. Rostrally, the basisphenoid contacts the ventral part of the laterosphenoid and terminates rostrally as a dorsoventrally tall cultriform process.

The temporal region of *Aegisuchus* bears a massive, rostrally-situated adductor tubercle and large, sub-horizontally oriented fossae for jaw closing muscles (Figs. 2, 4). The trigeminal foramen is large and bilobate, allowing the passage of the large divisions of the trigeminal nerve and bounded dorsally by the foramen for the supraorbital nerve. Both laterosphenoid bodies are robust and taper dorsally into large, mediolaterally-oriented capitate processes. Teardrop-shaped, isolated, vestigial epipterygoids [19] are sutured to both the left and right laterosphenoids. The dorsotemporal fossa is significantly reduced in size compared to other neosuchians.

### Brain volume, size and shape estimation

Volumetric reconstruction of the CT data revealed a brain cavity, substantially-sized middle ear cavity and eustachian system similar in morphology to that of extant crocodylians [20] (Fig. 4). Internal damage prohibited complete reconstruction of the inner and middle ear cavities, as well as much of the right side of the cranial cavity. The dorsum sellae was also damaged, preventing a clear estimation of the size and shape of the pituitary fossa. The olfactory bulbs were also missing and thus not included in the volume. Regardless, the complete reconstruction of the left side equaled ∼20 cm^3^. This value was doubled to result in an estimated complete endocranial volume equal to 40 cm^3^.

The regression equations describing the relationship between log cranial endocast volume (40 cm^3^) and log exoccipital-exoccipital width (∼23, Table 1) and log skull length (See Materials and Methods) estimated the skull length of *Aegisuchus* be between 2.08 and 2.86 m. Body length sizes estimated from regression analyses of these two skull lengths range between 15–21 m (*Gavialis*) and 16–22 m (*Crocodylus*). Even by our most conservative estimates, if *Aegisuchus* was 15 m long, it would still be significantly longer than the reported lengths for *Deinosuchus* (12 m [21]), *Gryposuchus*, (10 m [22]), *Purussaurus* (11–13 m [23]), and *Sarcosuchus* (∼11.65 m [4]). Admittedly, there is significant error associated with estimating skull and body size from a fragmentary, cranial specimen and a length of over 15 m is still almost certainly an overestimate. However, given the absolute size of the skull table and occipital condyle and the above results, *Aegisuchus* was undoubtedly a large animal. *Aegisuchus*, with a seemingly huge endocranial volume of 40 cm^3^, may simply have a larger relative endocranial volume compared to published data indicating that new, larger analyses of crocodyliform brain, and endocranial cavity size evolution is needed. *Aegisuchus* and its putative duck-faced relatives may have had much larger head length to body length ratio than other crocodyliforms; however, this seems unlikely given that other long-faced neosuchians, save gharials, typically show smaller head length to body length ratios [24]–[26].

**Table 1:** Available measurements of *Aegisuchus witmeri* ROM 54530 in cm.

Exoccipital-exoccipital width	∼23.0
Foramen magnum width	∼3.0
Cranial roof length	7.5
Cranial roof width	14.5
Occipital condyle width	4.5
Endocranial volume	∼40 cm^3^

Although most of the cranium is missing from *Aegisuchus*, the features of the braincase indicated the skull was likely flat and appears to be similar in profile to that reconstructed for *Stomatosuchus* [1], [27]. In dorsal view, the postorbital of *Aegisuchus* is anteriorly swept at about 52° from the midsagittal plane, compared to that of an adult *Alligator* (61°), the platyrostral basal brevirostrine *Leidyosuchus* (ROM 1903) (51°), and the short-snouted dyrosaur cf. *Rhabdognathus* (CNRST-SUNY [Centre National de la Recherche Scientifique et Technologique du Mali-Stony Brook University]-190), in which the process is almost vertically oriented (Fig. 5). In lateral view, the postorbital of *Aegisuchus* is anteriorly oriented at 147° from the skull table, 99° in *Alligator*, 125° in *Leidyosuchus*, and then posteriorly oriented 66° in *Rhabdognathus*. In lateral view, the quadrate is more posteriorly or horizontally swept relative to the skull table in *Aegisuchus* (164°) than in *Alligator* (150°) and *Leidyosuchus* (159°), whereas the quadrate is near vertical in *Rhabdognathus* (17°). In dorsal view, the quadrate again is swept caudally in *Aegisuchus* (40°) compared to *Alligator* (41°), *Leidyosuchus* (50°), and *Rhabdognathus* (107°). These data indicate that *Aegisuchus* was a very large, very flat-headed crocodyliform.

### Phylogenetic relationships

Our phylogenetic analyses (analysis of crocodyliforms [252 characters, 45 taxa]; analysis of Eusuchia [176 characters, 47 taxa]) (Figs. 7, 8, 9)(Supporting Information S1) found *Aegisuchus* and its sister taxon *Aegyptosuchus* to be derived eusuchians and the sister taxa of crown-clade Crocodylia. The close relationship between *Aegisuchus* and *Aegyptosuchus* to the exclusion of other taxa is supported by the following four unambiguous character states: no exposure of supraoccipital on skull table (82.1), capitate process of laterosphenoid oriented laterally (130.0), parietal and squamosal meet along caudal wall of dorsotemporal fossa (131.2), and postorbital suture oriented ventral to skull table (163.1). Aegyptosuchids are united with *Hylaeochampsa* and the crown-clade by one unambiguous character state: the possession of a basisphenoid which forms a thin sheet lateral to the basioccipital (113.1). Aegyptosuchids share one unambiguous character state with the crown-clade to the exclusion of *Hylaeochampsa*, a skull table with near horizontal sides (140.1). Finally, aegyptosuchids and several basal crocodylians (*Leidyosuchus*, *Eosuchus*) possess vestigial, isolated epipterygoids on their laterosphenoid bodies, further supporting the close phylogenetic relationship between the aegyptosuchids and crown crocodylians (Fig. 7) [19].

### Analysis 1 Results

The tree-space search found 8 most parsimonious trees (MPTs) with the following analytical parameters: length 1,002 steps, CI = 0.33, and RI = 0.64. *Aegisuchus* and *Aegyptosuchus* were found in a polytomy with the crown-clade crocodylians, suggesting that the former two taxa may be the nearest outgroup to the crown-clade or occupy an uncertain position within the crown. *Aegisuchus* and *Aegyptosuchus* share four unambiguous characters with eusuchians based on this data set (30.1, 59.0, 174.1, and 176.1) and share a close relationship to the exclusion of other taxa within the data set based on a multiple unambiguous characters (28.2, 56.1, 62.1, 69.1, 71.1, 72.1, 166.1, 169.1, and 175.0). Strong bootstrap scores exist for the Aegyptosuchidae (93%), but the interrelationships between higher neosuchians collapses except for a weakly supported ingroup of the crown-clade consisting of *Alligator*, *Leidyosuchus* and *Crocodylus* (59%) and a weakly supported longirostrine basal neosuchian clade consisting of *Dyrosaurus* and pholidosaurids (52%). Branch supports were weak through most internal nodes (decay index = 1), though very strong support (decay index = 3) was found for *Kaprosuchus*+*Mahajangasuchus*, the Thalattosuchia, and some internal nodes within the Notosuchia. Strong branch supports (decay index = 2) were found for the Aegyptosuchidae, Mesoeucrocodylia and Crocodyliformes. (Figs. 7–8).

### Analysis 2 Results

The tree-space search found that constraining *Aegyptosuchus*+*Aegisuchus* and *Laganosuchus* to be sister taxa produced substantially less parsimonious trees than the unconstrained tree (281 steps longer than the optimal trees). Interrelationships between mesoeucrocodylian clades were greatly collapsed in the strict consensus, although some higher neosuchian clades were better supported. Based on these results, a close relationship between *Laganosuchus* and *Aegisuchus*+*Aegyptosuchus* cannot be supported at this time. Although *Aegyptosuchus* shares several derived features with eusuchians, *Laganosuchus* shows many plesiomorphic character states in comparison to the Eusuchia, such as lacking a mandibular fenestra that is bordered by the dentary ventrally (181.0) and having a straight, posteriorly directed retroarticular process (208.0). It is worth noting that part of the type hypodigm of *Aegyptosuchus*, lost during World War II, showed the derived state for the latter character, as in other eusuchians, but unlike *Laganosuchus* as noted here. Despite the results of this analysis, it is still possible that these taxa are all closely related. Stromer's [1] description of the texture of the skull roof of *Stomatosuchus* closely resembles the characteristic texture found in *Aegisuchus* and *Aegyptosuchus*, and their respective braincases suggest them to have very large, very flat skulls, an additional similarity to *Stomatosuchus*. The mandible of *Laganosuchus* is very similar to that of *Stomatosuchus*, clearly allying these two. All of these features together suggest that, if aegyptosuchids and stomatosuchids are related, like many other transitional forms, these too have mosaic characters composed of primitive (e.g., mandible) and derived (e.g. braincase) features. However, additional diagnostic material is necessary to better understand the relationships of these taxa.

### Analysis 3 Results

The tree-space search found 12 MPTs with the following analytical parameters: length = 492 steps; CI = 0.46 and RC = 0.76. *Aegisuchus* and *Aegyptosuchus* share a close relationship to each other, to the exclusion of other taxa within the dataset (Figs. 7, 9). Further, the two genera were found to be the most derived stem-crocodylians in the data set among the resulting trees, if not possibly part of the crown-clade. Bootstrapping results suggest that the Aegyptosuchidae is strongly supported (92%), while the placement of the Aegyptosuchidae as the close outgroup to the crown-clade showed some support as well (62%). The crown-clade was not well supported to the exclusion of aegyptosuchids (<50%), but higher relationships within the crown-clade showed strong support (Gavialoidea, 81%; Alligatoroidea, 73%; and Crocodyloidea, 73%). Branch support was weak for most of Eusuchia, though Aegyptosuchidae had a strong branch support (decay index = 3), as did Crocodyloidea and its subnodes. This suggests that Aegyptosuchids may share some derived states found in one or more higher crown-clade ingroups, but that these putative relationships are not well supported. The position of aegyptosuchids as more derived than the outgroup taxa *Goniopholis* spp. and *Theriosuchus* spp. was supported by a single ambiguous character state (76.1). Aegyptosuchids were found to be more derived than *Bernissartia* based on a single ambiguous character state (92.1). One unambiguous character state unites aegyptosuchids with *Hylaeochampsa* and the crown-clade (113.1), whereas aegyptosuchids share two unambiguous character states with the crown-clade to the exclusion of *Hylaeochampsa* (140.1 and 168.1). The close relationship between *Aegisuchus* and *Aegyptosuchus* to the exclusion of other taxa is supported by the following four unambiguous character states (82.1, 130.0, 131.2, and 163.1).

Using the matrix of Sereno and Larsson (2009) [8] *Aegisuchus* differs from all other crocodyliforms in possessing the following unique combination of character states (Character.state): 67.2 posteroventral process of postorbital contacts quadrate; 18.2 quadrate ramus of pterygoid extends dorsally to laterosphenoid, does not form ventrolateral edge of trigeminal foramen; 28.2 raised rugose boss on dorsal surface of parietal. Using the matrix of Delfino et al. (2008) [18] *Aegisuchus* differs from all other crocodyliforms in possessing the following unique combination of character states (Character.state): 74.1 prootic laterally obscured by quadrate and laterosphenoid; 127.1 quadrate-pterygoid suture linear from basisphenoid exposure to the foramen ovale (as opposed to a significant ventral quadrate process on lateral braincase wall); 151.1 exoccipital sends a robust process ventrally in the basioccipital tuberosity.

## Discussion

Aegyptosuchids, and their alleged stomatosuchid relatives, are characterized by numerous features related to their extremely low-profile and inferred ambush-style predation [8]. The orbits lie flat upon the skull with no orbital ridges, and the thick skull table, robust laterosphenoids, inferred hypertrophied jaw-closing muscles (Fig. 4) and sub-horizontally oriented postorbital bars and quadrates suggest an animal with a slim lateral cranial profile. The enlarged exoccipital protuberances, rostrally-expanded epaxial musculature [28] and the massive occipital condyle indicate that the animal was adapted for strong jaw opening movements [29] compared to other crocodyliforms.

The extreme low profile of the cranium appears to be accompanied by shifts in jaw and cervical muscles. M. adductor mandibulae externus profundus is oriented more horizontally compared to that of other crocodyliforms, whereas the typically small m. adductor mandibulae externus medialis appears to be much larger relative to those of other crocodyliforms. The pronounced, rugose, rostrally-situated adductor tubercle and A-crest further indicates that the tendons and thus likely their attaching muscles, mm. adductor mandibulae externus superficialis and adductor mandibulae posterior, were well-developed.

Whereas the occipital surface faces caudally in most crocodyliform taxa, the dorsal half of the occipital surface in *Aegisuchus* faces caudodorsally and excavates portions of the paroccipital processes creating a broad shelf. This morphology suggests the presence of hypertrophied m. splenius capitis epaxial muscles, and, along with the giant exoccipital protuberances (attachments for m. transversus capitis [28]) and the large occipital condyle, suggests a capacity for strong vertical excursions. The lateral portions of the paroccipital process, and thus the cranial attachments of m. depressor mandibulae, are missing; however, the occipital emargination suggests this muscle was also large. Stromer [12] identified an articular with a long retroarticular process referable to *Aegyptosuchus*, and Sereno and Larsson [8] described a long, thin, laterally curved retroarticular process in the putative stomatosuchid *Laganosuchus thaumastos*, corroborating the inference of at least a large jaw opening muscle. *Aegisuchus*, with its inferred long, broad, flat face, would have been challenged to easily raise its head or open its mouth compared to smaller faced crocodyliforms, and in many ways these taxa converge upon facial morphology found among large-faced Triassic temnospondyl amphibians. Jenkins et al. [29] described a derived craniocervical articulation in *Gerrhothorax pulcherrimus* that facilitated head elevation and mouth opening by means of large, extended occipital condyles and a dorsally open—or soft-tissue-bounded— foramen magnum and rostral cervical vertebral column. Cervical anatomy is poorly known in these crocodyliforms specifically; however, the atlantal and axial neural arches are independently mobile and separate from the vertebral bodies in crocodyliforms, thereby achieving a similar open, flexible dorsal vertebral column at the craniocervical junction. Thus, the expanded attachments of epaxial muscles, large bulbous occipital condyle, and m. depressor mandibulae would facilitate head elevation in *Aegisuchus* to a degree not found in other crocodyliforms.

Drawing from a small fragment of preserved gular skin, Nopcsa [27] interpreted *Stomatosuchus* to be a suspension feeder, relying on a large pouch suspended between putatively edentulous mandibles. Stromer [12] found the specimen's preservation to be too questionable to decisively infer toothlessness. Sereno and Larsson [8] proposed a piscivorous diet for the toothed *Laganosuchus*, a putative sister taxon of *Stomatosuchus*. The known ichthyofauna of the Kem Kem Formation might support this, because aegyptosuchids could have potentially preyed on slow moving fish such as coelacanths, lungfish and bichirs [16] as well as opportunistically on terrestrial vertebrates.

*Aegisuchus* lends additional insight into the evolution of the orbitotemporal region, which was quite disparate among crocodyliform clades during the Cretaceous [20]. Although a comprehensive analysis of character evolution is in progress (Holliday et al., unpubl. data), the presence of isolated epipterygoids on the bodies of the laterosphenoids supports the systematic placement of this species within Eusuchia. Several basal members of the clade (*Leidyosuchus*, *Eosuchus*) as well as close outgroup taxa (*Goniopholis*, *Eutretraunosuchus*) also possess similarly shaped, vestigial epipterygoids and lack laterosphenoid lateral bridges like *Aegisuchus* [19]. *Aegisuchus*, however, does not have a laterosphenoid lateral bridge. These features suggest the regression, and eventual disappearance of this important feature of the primitive reptilian palate was almost complete in derived eusuchians.

The most peculiar feature of *Aegisuchus* is the inferred vasculature emerging from the dorsotemporal fossae which then circumscribes the characteristic central boss on the skull table. Virtually all crocodyliforms have skull tables marked by dimples and rugosities, and some develop pronounced postorbital and squamosal horns, such as *Crocodylus rhombifer* and *Voay robustus* [30]. However, none have the peculiar, centralized structure whose texture suggests a more thickened, dermal attachment [31] than normally found. The skull table of *Aegyptosuchus* is also rough and marked by a centralized boss, but, a clear vascular fossa is not evident; suggesting this particular feature may be transient or variable among aegyptosuchids (Figs. 3, 4). However, whether the vascular bed is directly related to the integumentary boss on the skull table is difficult to determine. Neosuchians do not have large cervical osteoderms that border the skull roof, thus negating the inference of a cervical armored region in need of additional vascular supply. Crocodyliforms do not possess known glandular material, such as salt glands on their skull roof, thus negating vascular supply for these tissues. The hypertrophied craniocervical muscles may have required additional vasculature, but there are no evident vascular correlates communicating between the dorsotemporal fossae and occipital surface. Although speculative, potential roles for vascularized tissues on the skull roof include a potential for thermoregulatory adaptation given the temporoorbital vessels communicate with the encephalic vessels and ophthalmic rete [32]. Regardless, the structure of the central boss and surrounding tissues are well placed to serve as a display structures. During territorial and mating displays alligatorids and crocodylids both raise their heads out of the water as a means of communication, including head slapping and snout-lifting behaviors which increase the profiles of their heads [33]. We envision *Aegisuchus* employing similar behaviors while also displaying its raised, eyespot-like boss on its skull table.

The discovery of *Aegisuchus* from the early Late Cretaceous of Africa potentially challenges orthodox behavioral assumptions of crocodyliforms as well as biogeographical models of eusuchian evolution. This time period marked an important transitional time during the breakup of the northern hemisphere Laurasia from Africa and West Gondwana due to the expanding Tethyan seaway and Atlantic Ocean [15]. Current biogeographical and phylogenetic hypotheses suggest eusuchian evolution occurred largely on the Laurasian supercontinent during the Early Cretaceous, and the origin of Crocodylia to likely have been in Late Cretaceous North America [15]. However the discovery of a closely-related African clade of derived aegyptosuchid eusuchians casts new light on this scenario, indicating the origins of Crocodylia may instead be from the Tethyan region. Capable of emigrating long distances, aquatic crocodyliforms are notoriously geographically dispersive [6], [14]; however, the derived features of *Aegisuchus* and its kin as well as the lack of similar taxa elsewhere suggest that these predators were endemic to Late Cretaceous northern Africa, where they filled a derived, behaviorally-distinct, macropredatory niche among a diverse and mature vertebrate fauna.

## Materials and Methods

### Scanning methods, size and shape estimation

The Royal Ontario Museum granted access for the specimen to be studied. The type specimen was medical CT-scanned helically on a General Electric (GE) LightSpeed VCT CT scanner at Cabell Huntington Hospital, Huntington, WV at 625 µm slice thickness 120 kV and approximately 300 mA. Data are archived with the authors and at the Royal Ontario Museum. The DICOM data were imported into Amira 4.1.2 (Mercury-TGS, Chelmsford, MA) for viewing, analysis, and reconstruction. To estimate brain volume, the entire available cranial cavity between the foramen magnum and orbit was manually segmented, and the resulting incomplete endocast was divided along the mid-sagittal plane and then doubled to equal total endocast volume (Fig. 4). Endocast volume and skull table measurements were included in published regression analyses: brain volume (*x*) (y = 1.45x+0.39) [8] and skull table width (i.e., exoccipital-exoccipital width or *x*)(y = 0.96x+0.407) [23] and an unpublished dataset consisting of 9 *Alligator mississippiensis* specimens (y = 150x+0.40) [34] to estimate skull length (*y* for all regressions). These two skull lengths (*x*) were used to estimate total body length (*y*) using the regression analyses by Sereno et al. [4] for *Crocodylus porosus* (y = −20.224+7.717x) and *Gavialis gangeticus* (y = −69.369+7.4x). To illustrate skull flatness, best fit lines from the skull table midline through the postorbital and quadrate/paroccipital processes were drawn in dorsal and lateral views in Amira 4.1.2 to estimate their angles in 3D reconstructions of *Aegisuchus* and a subsample of fossil crocodyliform skulls: cf. *Rhabdognathus* (CNRST-SUNY-190) and *Leidyosuchus canadensis* (ROM 1903) [17] (Fig. 5).

### Nomenclatural Acts

The electronic version of this document does not represent a published work according to the International Code of Zoological Nomenclature (ICZN), and hence the nomenclatural acts contained in the electronic version are not available under that Code from the electronic edition. Therefore, a separate edition of this document was produced by a method that assures numerous identical and durable copies, and those copies were simultaneously obtainable (from the publication date noted on the first page of this article) for the purpose of providing a public and permanent scientific record, in accordance with Article 8.1 of the Code. The separate print-only edition is available on request from PLoS by sending a request to PLoS ONE, Public Library of Science, 1160 Battery Street, Suite 100, San Francisco, CA 94111, USA along with a check for $10 (to cover printing and postage) payable to “Public Library of Science”.

In addition, this published work and the nomenclatural acts it contains have been registered in ZooBank , the proposed online registration system for the ICZN. The ZooBank LSIDs (Life Science Identifiers) can be resolved and the associated information viewed through any standard web browser by appending the LSID to the prefix “http://zoobank.org/”. The LSID for this publication is: urn:lsid:zoobank.org:pub:8D31134D-25B4-4357-9FE8-C74DC51735BC

### Phylogenetic analyses

We investigated the systematic position of *Aegisuchus witmeri* using two datasets designed to illuminate its relationship among crocodyliforms [8] and eusuchians [18], respectively (Supplementary Info). The three analyses, including an additional crocodyliform analysis constraining an aegyptosuchid and stomatosuchid clade: aegyptosuchids+stomatosuchids within Crocodyliformes were run using the following search parameters:

### Analysis 1: The relationships of *Aegisuchus* within Crocodyliformes

In order to infer the position of aegyptosuchids and confirm the monophyly of *Aegyptosuchus* with *Aegisuchus*, both taxa were incorporated into an existing global data set for crocodyliforms. Although at least three large global data sets currently exist, the matrix which offers the best taxon representation for North African Cretaceous crocodyliforms, Sereno and Larsson [8], was used. *Aegyptosuchus* was scored only from braincase and skull table characters which could be directly observed. The parameters of the data set as defined by Sereno and Larsson [8] were not altered, aside from adding a new character state to character 28 to reflect the derived character state found in both *Aegyptosuchus* and *Aegisuchus* (i.e., a raised boss on the dorsal surface of the parietal). All other prior assumptions were unchanged, including character ordering. Delayed transformation (DELTRAN) character optimization was applied, but only unambiguous characters were considered for assessing the underlying support of each node.

The data set was subjected to a tree-space search using PAUP 4.0b10 [35] The heuristic search was employed using 1,000 random addition sequences (addseq = random, nreps = 1000) with three trees sampled per iteration (nchuck = 3, chuckscore = 1), then all trees found by this procedure were then branch-swapped again using tree bisection-reconnection (TBR) to check for shorter resolutions and to fill out tree space. Bootstrapping was carried out to assess the robustness of the resulting trees using 1,000 randomized (with replacement) character samples, with each bootstrap sample using heuristic search with 10 random addition sequences (addseq = random, nreps = 10) with 10 trees sampled per iteration (nchuck = 10, chuckscore = 1). Decay indices ( = “branch support” of Bremer, 1994 [36]) were calculated for all internal branches using treespace searches that retained suboptimal nodes.

### Analysis 2: Are aegyptosuchids related to the putative “stomatosuchid” *Laganosuchus*?

Carroll [37] suggested that *Aegyptosuchus* may have had close affinities with the Stomatosuchidae, but this hypothesis was never rigorously tested. Because the only known material of *Stomatosuchus* was destroyed, direct comparison with aegyptosuchids was impossible. However, new putative stomatosuchid material (*Laganosuchus*) was recently described [8]. To test whether a close relationship between *Aegisuchus*, *Aegyptosuchus* and the stomatosuchids was a possibility, *Aegisuchus*+*Aegyptosuchus* were constrained with *Laganosuchus* as sister taxon to the exclusion of other higher neosuchians.

### Analysis 3: The relationships of *Aegisuchus* with Eusuchia

Recognizing from the first analysis that aegyptosuchids appear to show a strong affinity towards the crown-clade and eusuchian crocodyliforms, testing the relationships between aegyptosuchids with these taxa in a larger data set was warranted. Therefore, a third analysis was performed using a recently published data set for testing the interrelationships between crown-clade crocodylians and their immediate outgroups [18] using the same parameters listed in Analysis 1.

### Geographic and stratigraphic ranges of higher neosuchians

The geographic and stratigraphic ranges of higher neosuchians (Fig. 6) were derived from various literature sources, but predominantly from Salisbury et al. [14] and Brochu [38]–[39]. Additional data for the stratigraphic range of *Theriosuchus* was supplemented by new information regarding *T. sympiestodon* from the Maastrichtian of Romania [40]. We recognize fragmentary material from Thailand has been referred to *Theriosuchus* [41], but barring further investigation are cautious about this referral, and did not include this data for determining the geographic range of this taxon.

### Phylogenetic definitions: Aegyptosuchidae Kuhn, 1936

#### Diagnosis

Large-sized eusuchians with raised, rugose integumentary boss on the dorsal surface of the parietal; parietal and squamosal meet along caudal wall of dorsotemporal fenestra; parietal not completely excluded from the occipital surface; supraoccipital is not exposed on dorsal skull table; laterosphenoid capitate processes are laterally oriented; ventral edge of squamosal groove for external ear flap musculature is lateral to the dorsal edge; squamosal posteromedial branch is posterolaterally oriented; depression for palpebral on anterodorsal surface of the prefrontal; paroccipital processes of squamosal bear a long process lateral to the cranioquadrate opening; postorbital bears a prominent anterolateral projection distinct from its dorsal corner; external auditory meatus fossa does not extend further than the posterior margin of the postorbital; postorbitosquamosal suture passes medially to the ventral skull table.

#### Etymology

Aegyptosuchidae was established as a family name on the basis of *Aegyptosuchus peyeri* Stromer 1933 [12].

#### Phylogenetic definition

The least inclusive clade containing *Aegisuchus witmeri* n. gen. n. sp. and *Aegyptosuchus peyeri* Stromer 1933, as long as it does not include *Alligator mississippiensis* (Daudin 1802), *Bernissartia fagesii* Dollo 1883 *Crocodylus niloticus* Laurent 1768, *Gavialis gangeticus* (Gmelin 1789), *Hylaeochampsa vectiana* (Owen 1874), or *Susisuchus anatoceps* (Salisbury, Molnar, Frey and Willis 2003).

## Supporting Information

Supporting Information S1 Character lists and matrices used in phylogenetic analysis.
(DOC)
