# Supplementary material for: A New Eusuchian Crocodyliform with Novel Cranial Integument and Its Significance for the Origin and Evolution of Crocodylia
Source: PLoS One. 2012 Jan 31;7(1):e30471. doi: 10.1371/journal.pone.0030471 (PMC3269432; doi:10.1371/journal.pone.0030471)
Supplement: Supporting Information S1 — Character lists and matrices used in phylogenetic analysis. (DOC) [file pone.0030471.s001.doc]

**Supplementary Information**

**A new eusuchian crocodyliform with novel cranial integument and its significance for the origin and evolution of Crocodylia**

**CM Holliday and NM Gardner**

S1: Data matrix and character list

**Character List used in Analyses**

**Analyses 1 & 2:** Character list from Sereno and Larsson (2009)[5]. Character numbers and ordering assumptions follow their original. See Sereno and Larsson (2009) for further discussion on character ontologies and references.

1. External surface of dorsal cranial bones: relatively smooth (0) or slightly grooved (1) or heavily ornamented with deep pits and grooves (2).
2. Snout lateral expansion at orbits: gradual (0) or abrupt (1).
3. Snout length (anterior margin of orbits to rostrum) relative to remainder of skull: equal or longer (0) or shorter (1).
4. Snout cross-section dimensions: higher than wide (0) or equally high as wide (1) or wider than high (2).
5. Antorbital fenestra size relative to orbit: about half (0) or smaller than half but present (1) or only an external fossa (may have a tiny fenestra) (2). Ordered.
6. Shape of antorbital fossa: subcircular or subtriangular (0) or elongated, low, and oriented obliquely (1).
7. Anteroposterior length of supratemporal fenestrae: equal to or shorter than orbits (0) or much longer than orbits (1).
8. Nasal extension dorsally into external nares: absent by maxilla-maxilla contact (0) or absent by premaxilla-premaxilla contact (1) or none but contacts external nares (2) or present and less than 50 perfect (3) or present and 50 percent or more but not completely (4). Ordered.
9. Dorsal surface of rostrum: curves smoothly (0) or bears medial boss (1).
10. Nasal-nasal suture: unfused (0) or partially or completely fused (1).
11. Nasal, posterior tip: converge at sagittal plane (0) or separated by an anterior sagittal projection of frontals (1).
12. Posterolateral region of nasals: flat surface facing dorsally (0) or lateral region deflected ventrally, forming part of the lateral surface of the snout (1).
13. Immediate preorbital region cross section: squared (0) or gently curved (1).
14. Prefrontal and lacrimal orbital margin: flat (0) or dorsally upturned to telescope orbit (1).
15. Prefrontals anterior to orbits: elongated, parasagittal orientation (0) or short, broad, and oriented anterolaterally (1).
16. Orbital margin of prefrontal: confluent with orbit (0) or projects laterally (1).
17. Prefrontal and lacrimal border to orbit: flat, confluent to snout surface (0) or invaginated, forming elevated rims (1).
18. Depression on prefrontal for a palpebral element: absent (0) or thin groove (1) or deep groove terminating anteriorly in a deep fossa (2). Ordered.
19. Transverse external prefrontal-frontal ridge: absent (0) or present and complete over prefrontals and frontals (1).
20. Prefrontal descending process: no palatine contact (0) or cylindrical or thin anteroposterior suture with palatine (1) or transversely broad suture with palatine (2).
21. Lacrimal-nasal contact: broad (0) or partially separated by posterior process of maxilla or (2) absent (maxilla separates lacrimal and nasal). Ordered.
22. External lacrimal shape: longer than broad (0) or nearly as broad as long (1).
23. Total lacrimal length relative to total prefrontal length: longer (0) or subequal (1) or shorter (2).
24. Anterior ramus of frontals relative to anterior ramus of prefrontals: posterior (0) or anterior (1).
25. Ventral half of lacrimal: extends posteroventrally to widely contact jugal (0) or tapering posteroventrally to not or only slightly contact jugal (1).
26. Frontal-frontal contact: paired (0) or fused (1).
27. Width of frontals between orbits relative to mid-length width across nasals: narrow -similar to width of nasals (0) or broad - about twice the width of nasals (1).
28. Dorsal surface of frontal and parietal: flat (0) or with sagittal ridge (1).
29. Frontal orbital margin: flat (0) or dorsally upturned (1).
30. Frontoparietal suture entry into supratemporal fenestra: deep, preventing broad postorbital (or postfrontal) - parietal contact (0) or no entry, broad postorbital (or postfrontal)-parietal contact (1).
31. Palpebrals: absent (0) or one small present (1) or one or multiple present and largely covering the dorsal surface of the orbit (2).
32.  Dermal bone overhang about the supratemporal fenestra: absent (0) or present only medially (1) or present about all but the anteromedial corner (fossa) (2). Ordered.
33. Medial borders of supratemporal fenestrae: separated by a broad sculptured region (0) or separated by a thin sculpted region (1) or contact to form a low sagittal crest (2).
34. Medial dorsal edges of supratemporal fenestrae: flat (0) or raised (1). Ordered.
35. Posterior extent of orbital edge of jugal: confluent with postorbital bar (0) or displaced laterally and ends anterior to postorbital bar (forming posteroventral notch in orbit) (1) or displaced laterally and ends at or just behind postorbital bar (2) or displaced laterally and ends near posterior corner of infratemporal fenestra (3).
36. Width of anterior process of jugal relative to posterior process: subequal (0) or about twice as broad (1). Ordered.
37. Dorsal surface of jugal beneath infratemporal fenestra: ovate cross-section (0) or longitudinal crest (1).
38. Anterior process of jugal relative to infratemporal fenestra anteroposterior length: subequal (0) or much longer (1).
39. Anterior margins of lacrimal and jugal: confluent with no notch at anterior contact (0) or jugal edge convex producing an anterior notch at contact (1).
40. Jugal participation in margin of antorbital fossa: present (0) or absent (1).
41. Lateral surface of anterior process of jugal: flat or convex (0) or broad shelf below orbit with triangular depression beneath (1).
42. Jugal postorbital process base projection: posterodorsal (0) or dorsal (1) or anterodorsal (2).
43. Jugal anterior margin relative to orbit: not anterior to (0) or anterior to (1).
44. Jugal ventral margin: relatively straight (0) or gentle concave arch (1) or steep concave peak at level of postorbital bar (2). Ordered.
45. Large foramen on the lateral surface of jugal, near its anterior margin: absent (0) or present (1).
46. Lateral surface of jugal-ectopterygoid contact: inset from lateral jugal margin (0) or confluent with lateral jugal margin forming a depression (1).
47. Jugal posterior process exceeds posteriorly the infratemporal fenestra: yes (0) or no (1).
48. Quadratojugal-postorbital contact: absent (0) or narrows dorsally and contacts a small region of the postorbital (1) or broadens dorsally to contact most of the postorbital bar to diminish the infratemporal fenestra (2). Ordered.
49. Spina quadratojugalis: absent (0) or small or low crest (1) or prominent (2). Ordered.
50.  Elements at posterior angle of infratemporal fenestra: quadratojugal (0) or quadratojugal and jugal (1) or jugal (2).
51. Quadratojugal posteroventral extension: does not reach quadrate condyles (0) or reaches but does not participate in quadrate condyles (1) or forms lateral extension to the quadrate condyles and participates in mandibular joint (2). Ordered.
52. Length of anterior process of quadratojugal: short or absent (0) or long (less than half length of lower temporal bar) -- moderate (1/3 of lower temporal bar) (1) or long (greater than half of lower temporal bar (2). Ordered.
53. Quadratojugal ornamentation at its base: absent (0) or present (1).
54. Posterior skull table: non-planar (squamosal ventral to horizontal level of postorbital and parietal) (0); planar (postorbital, squamosal and parietal on same horizontal plane) (1).
55. Cranial table width relative to ventral portion of skull: nearly as wide (0) or narrower (1).
56. Dorsal and ventral edges of squamosal groove for external ear valve musculature: absent (0) or ventral edge is lateral to dorsal (1) or ventral edge is directly beneath dorsal (2).
57. Posterior region of auditory fossa: opens posteriorly (0) bounded posteriorly by a posteroventrolateral extension of the squamosal and exoccipital (1).
58. Squamosal prongs: short or absent (0) or present, depressed from skull table, unsculpted (1) or present, level with skull table, sculpted (2) or present, upturned, sculpted (3).
59. Distal squamosal prong: tapered (0) or broad (1).
60. Posterolateral overhanging rim of supratemporal fossa: absent, anterior opening of temporo-orbital foramen visible in dorsal view (0) or present and temporo-orbital foramen partially occluded from dorsal view (1).
61. Squamosal posterolateral region, lateral to paroccipital process: narrow (0) or bearing a subrounded flat surface (1).
62. Posteromedial branch of squamosal orientation: transverse (0) or posterolateral (1).
63. Parietal dorsal surface between supratemporal fenestrae: broad sculpted region (0) or sagittal crest (1).
64. Postorbital participation in infratemporal fenestra: nearly or completely excluded (0) or present (1).
65. Postorbital bar sculpturing (if skull sculpted): present (0) or absent (1).
66. Postorbital bar: transversely flattened (ectopterygoid does not strongly contact bar) (0) or massive (roughly anterolateral elliptical cross-section) (1) or slender (cylindrical); roughly anteromedially elliptical (2).
67. Postorbital posteroventral process: absent (0) or present as a thin descending process from the postorbital along the quadratojugal (1) or present and contacts the quadrate (2). Ordered.
68. Anterolateral projections on postorbital bar: absent (0) or present (1).
69. Anterior extension of external auditory meatus fossa: squamosal (0) or onto posterior margin of postorbital, separated from anterior margin by a vertical ridge (postorbital roof overhangs postorbital-squamosal suture) (1) or to anterolateral edge of postorbital (2) or along entire length of postorbital and continues into orbit over a thin ramus of the postorbital (3). Ordered.
70. Vascular opening on lateral edge of dorsal part of postorbital bar: absent (0) or present (1).
71. Postorbital with prominent anterolateral projection distinct from dorsal corner: absent (0) or present (1).
72. Depression on anterodorsal surface of postorbital for a palpebral element: absent (0) or present (1).
73. Postorbital bar relative to dorsolateral edge of postorbital: continuous (0) or inset medially (1).
74. Bar between orbit and supratemporal fossa: broad (0) or narrow (fossa covers entire bar) (1).
75. Position of postorbital relative to jugal on ventral end of postorbital bar: anterior (0) or medial (1) or lateral (2).
76. Postorbital-ectopterygoid contact: present (0) or absent (1).
77. Bones on lateral surface of postorbital bar: postorbital and jugal (0) or only postorbital (1).
78. Premaxillary labial process extending anteriorly beyond tooth row: absent (0) or present (1).
79. Premaxilla midline extension into anterior margin of external nares: none (0) or small projection (less than 10 percent of length of nares) (1) or present and less than 50 percent (2) or present and more than 50 percent but not completely (2). Ordered.
80. Premaxilla midline extension from posterior margin of external nares: absent (0) or present and thick (1) or present and thick to form a posterodorsal notch (2). Ordered.
81. External nares orientation: lateral (0) or dorsal (1) or anterior or anterolateral (2).
82. Circumnarial fossa: absent (0) or present (1).
83. Single or paired foramina at posterolateral corner of premaxilla above tooth row: absent (0) or present (1).
84. Foramen on palatal premaxilla-maxilla contact near tooth row: small or absent (0) or large (1) or large and connects with an elongate foramen in the external premaxilla-maxilla suture immediately above the tooth line (2).
85. Premaxilla palatal shelves: do not meet posteriorly (0) or meet posteriorly (1).
86. Incisive foramen: present and large (length equal to or more than half the greatest width of premaxillae) (0) or present and small (length less than half the width of the premaxillae) (1) or absent (palatal parts of premaxillae in contact along entire length) (2).
87. Premaxilla tooth count: two (0) or three (1) or four (2) or five (3). Ordered.
88. Anterior two premaxillary teeth: separate (0) or nearly confluent (1).
89. Posterodorsal premaxillary process extension: absent (0) or present but not beyond third maxillary alveolus (1) or present and beyond third maxillary alveolus (2). Ordered.
90. Premaxilla-maxilla lateral fossa excavates alveolus of last premaxillary tooth: no (0) or yes (1).
91. Premaxilla-maxilla suture in palatal view, medial to alveolar region: anteromedially directed (0) or sinusoidal, posteromedially directed on its lateral half and anteromedially directed along its medial region (1) or posteromedially directed (2).
92. Deep fossa between and behind first and second premaxillary teeth to accommodate an enlarged, procumbent first dentary tooth: absent (0) or present (1).
93. Ventral edge of premaxilla location with respect to ventral edge of maxilla: same height (0) or ventral (1).
94. Premaxillary palate circular paramedian depressions: absent (0) or present located anteriorly on the premaxilla (1) or present located at the premaxilla-maxilla suture (2).
95. Procumbent premaxillary alveoli: absent (0) or present (1).
96. Premaxillary anterior alveolar margin orientation: vertical (0) or inturned (1).
97. Premaxillary tooth row orientation: arched labially from midline (0) or angled posterolaterally, at 120 degree angle (1) or set in a relatively straight posterolateral orientation (2).
98. Last premaxillary tooth position to first maxillary tooth: anterior (0) or anterolateral (1).
99. Premaxillary and anterior dentary tooth row orientation: posterolateral (0) or nearly transverse (1).
100. Penultimate posterior premaxillary tooth size relative to anterior premaxillary teeth: similar (0) or much longer (1).
101. Anteormedial extension of incisive foramen: far from premaxillary tooth row (level of second or third alveolus) (0) or abuts premaxillary tooth row (1).
102. Wedge-like anterior process of maxilla on lateral surface of premaxilla-maxilla suture: absent (0) or present (1).
103. Enlarged anterior dentary teeth occlusion at premaxilla-maxilla suture: enlarged teeth absent (0) or lingually within an internal fossa (fossa may extend dorsally to form a foramen) (1) or labially within a laterally open notch (2).
104. Sculpturing along alveolar margin on lateral surface of maxilla: absent (0) or present (1).
105. Maxilla-maxilla contact on palate: only posterior ends not in contact at sutures with palatines (0) or complete (1).
106. Ventrolateral edge of maxilla in lateral view: straight (0) or single convexity (1) or double convexity (“festooned”) (2). Ordered.
107. Posterior extent of maxilla: posterior to anterior margin of orbit (0) or anterior to anterior margin of orbit (1).
108. Maxillary depression (separate from antorbital fenestra) on lateral surface near lacrimal: absent (0) or present (1).
109. Sagittal torus on maxillary palatal shelves: absent (0) or present (1).
110. Longitudinal depressions on palatal surface of maxilae and palatines: absent (0) or present (1).
111. Large and aligned neurovascular foramina on lateral maxillary surface: absent (0) or present (1).
112. Maxillary tooth number: 10 or more (0) or less than 10 (1).
113. Posterior maxillary and dentary teeth implantataion: thecodont (0) or within an incompletely divided alveolar groove (1).
114. Ornamentation on carinae of maxillary and opposing dentary teeth: smooth (0) or serrations (1) or denticles (2).
115. Compressed crown of maxillary teeth orientation: parallel to longitudinal axis of tooth row (0) or obliquely disposed (1).
116. Maxillary teeth lateral compression: absent (0) or present (1).
117. Position of first enlarged maxillary teeth: maxillary teeth relatively  homodont (0) or second or third alveoli (1) or fourth or fifth alveoli (2).
118. Tooth carinae: absent or smooth or crenulated (0) or denticulate (1).
119. Cheek teeth crown bases: not constricted (0) or constricted (1).
120. Vomer palatal exposures: present (0) or absent (1).
121. Palatine secondary palate: palatines form palatal shelves that do not meet (0) or form palatal shelves that meet along anterior 2/3 of secondary palate (posteriorly open V may be filled by pterygoids) (1) or palatal shelves meet along their entire length (linear palatine-pterygoid contact) (2). Ordered.
122. Palatine-pterygoi suture on secondary palate relative to posterior angle of suborbital fenestra: nearly at (0) or far from (1).
123. Posterolateral edges of palatines on secondary palate: parallel (0) or flare laterally to form a shelf (1).
124. Anterior process of palatine on secondary palate: pointed (0) or rounded (1) or wide and squared (flat anteriorly) (2).
125. Palatine-pterygoi contact on palate: palatine overlies pterygoid (0) or palatine firmly sutured to pterygoid (1).
126. Pterygoid secondary palate: absent (0) or thin shelf that does not meet (1) or secondary palate with anterior margin of choanae located in anterior one-half of pterygoid (2) or secondary palate with anterior margin of choanae located in posterior one-half of pterygoid (3). Ordered.
127. Choanae projection: posteroventrally into a midline depression continuous with pterygoid surface (0) or posteriorly walled by pterygoids (1) or posteriorly walled by pterygoids with a ventrally raised posterior rim (2). Ordered.
128. Paired anterior palatal fenestra: absent (0) or present (1).
129. Palatine anterior palatal fenestra: absent (0) or present (1).
130. Posterior pterygoid processes: absent or low ridges (0) or present and near level of palate (1) or present and tall (2).
131. Posteromedial region of pterygoid in occipital aspect: not visible (0) or visible but less than basioccipital height (1) or visible and subequal in height to basioccipital (2). Ordered.
132. Combined width of pterygoids in palatal aspect: not more than twice wider than long (0) or more than twice wider than long (1). Ordered.
133. Depression on primary pterygoid palatae posterior to choana: absent or moderate in size, narrower than palatine bar (0) or wider than palatine bar (1).
134. Primary pterygoid palate: forms posterior half of choanal opening (0) or forms posterior, lateral and part of the anterior margin of the choana (1) or completely enclose choana (2). Ordered.
135. Pterygoid-pterygoi contact on primary palatal plane: completely to basipterygoid processes (but open posteriorly to form a V over basisphenoid) (0) or complete with basisphenoid length approximately 1/3 width (1) or complete with basisphenoid nearly hidden by a near pterygoid-basioccipital contact (2). Ordered.
136. Anterior edge of choanae location with respect to posterior margin of suborbital fenestrae: at or anterior to (0) or posterior (1).
137. Quadrate process of pterygoid: well developed (0) or poorly developed (1).
138. Pterygoid flanges: laminar and expanded (0) or bar-like (1).
139. Quadrate ramus of pterygoid: extends dorsally to laterosphenoid (0) or extends dorsally to laterosphenoid and forms ventrolateral edge of trigeminal foramen (1).
140. Quadrate ramus of pterygoid in ventral aspect: broad (0) or narrow (1).
141. Pterygoid flanges: thin and laminar (0) or dorsoventrally thick, with pneumatic spaces (1).
142. Choanal groove: undivided (0) or partially separated (1) or completely separated (2). Ordered.
143. Choanal septum shape: narrow vertical bony sheet (0) or T-shaped bar expanded ventrally (1).
144. Choanal septum, ventral surface: smooth to slightly depressed (0) or marked by an acute gorove (1).
145. Ectopterygoid projection medially on ventral surface of pterygoid flange: minimal (0) or broad, extending approximately over the lateral half of the pterygoid flange (1).
146. Ectopterygoid medial process: single (0) or forked (1).
147. Ectopterygoid-maxilla contact: absent (0) or present but ectopterygoid only abuts maxilla (1) or present and ectopterygoid nears maxillary tooth row (2) or present and broadly separated from tooth row by maxilla (3). Ordered.
148. Ectopterygoid, relation to postorbital bar: no support (0) or contributes to base of bar(1).
149. Ectopterygoid extension along lateral pterygoid flange: not to posterior tip of pterygoid (0) or to posterior tip of pterygoid (1).
150. Posterior ectopterygoid process along ventral surface of jugal: absent (0) or very small (1).
151. Quadrate body orientation distal to otoccipital-quadrate contact in posterior view: ventral (0) or ventrolateral (1).
152. Cross section of distal end of quadrate: mediolaterally wide and anteroposteriorly thin (0) or subquadrangular (1).
153. Quadrate condyles: witih poorly developed intercondylar groove (0) or medial condyle expands ventrally, separated from the lateral condyle by a deep intercondylar groove (1).
154. Quadrate distal end: with only one plane facing posteriorly (0) or with two distinct faces in posterior view, a posterior one and a medial one bearing the foramen aerum (1).
155. Quadrate major axis orientation: posteroventral (0) or ventral (1) or anteroventral (2).
156. Posterior edge of quadrate body: broad medial to tympanum, gently concave (0) or posterior edge narrow dorsal to otoccipital contact, strongly concave (1).
157. Squamosal-quadrate contact within the otic aperture to posteriorly bound the external auditory meatus: absent (0) or present with a smooth posteroventral margin bordering the otic aperture (1) or present with a posteroventral notch in the contact (2).
158. Quadrate-squamosal-otoccipital contact to enclose cranioquadrate space: absent (0) or present near lateral edge of skull (1) or present with quadrate-squamosal contact broad laterally (2). Ordered.
159. Prominent crest on dorsal surface of distal quadrate that extends proximally to lateral extent of quadrate-exoccipital contact: absent (0) or present (1).
160. Preotic siphonal foramina: absent (0) or single (1) or three or more (2). Ordered.
161. Dorsal primary head of quadrate contact: only squamosal (0) or squamosal and (or near) laterosphenoid (1).
162. Quadrate-basisphenoi contact: dorsolateral contact (0) or dorsolateral and anterolateral contact (1).
163. Distal quadrate relative to quadrate body: distinct (0) or indistinct ventromedial contact of quadrate body with otoccipital (1).
164. Jaw articulation (quadrate condyle), position relative to maxillary tooth row: above or near level (0) or below (1).
165. Laterosphenoid bridge: absent (0) or at least partially complete (1).
166. Prominent boss on paroccipital process: absent or reduced, with short process lateral to cranioquadrate opening (0) or present, with long process lateral to cranioquadrate opening (1).
167. Ventromedial portion of exoccipital adjacent to basioccipital tubera: slender (0) or hypertrophied (1).
168. Large ventrolateral region of paroccipital process: present (0) or absent (1).
169. Supraoccipital exposure on dorsal skull table: absent (0) or small, parietal still reaches portion of the occipital surface (1) or large, parietal excluded from occipital surface (2). Ordered.
170. Mastoid antrum: extending into a fossa in supraoccipital (0) or extends through a complete transverse canal in supraoccipital (1).
171. Otoccipital large ventrolateral part ventral to paroccipital process: absent (0) or present (1).
172. Basioccipital and ventral part of otoccipital orientation: posteroventrally (0) or posteriorly (1).
173. Lateral Eustachian tube openings: located posterior to medial opening (0) or aligned anteroposteriorly and dorsoventrally (1).
174. Basisphenoid lateral exposure on braincase: absent (0) or present (1).
175.  Laterosphenoid, capitate process orientation from midline: lateral (0) or anteroposterior (1).
176. Posterior surface of supraoccipital: nearly flat (0) or bilateral posterior prominence (1).
177. Basioccipital tuberosity: poorly developed (0) or large and pendulous (1).
178. Mandibular symphysis, terminal orientation: horizontal or slightly anterodorsal (0) or anterodorsally at approximately 45 degrees at a distinct angle from jaw line (1).
179. Mandibular symphysis shape in lateral view: shallow and tapering anteriorly (0) or deep and tapering anteriorly (1) or deep and anteriorly convex (2) or shallow and anteriorly convex (3).
180. Dorsal surface of mandibular symphysis: flat or slightly concave (0) or strongly concave and narrow, trough shaped (1).
181. Dentary extension beneath mandibular fenestra: present (0) or absent (1).
182. Anterior caniniform dentary tooth near third position: absent (0) or present (1).
183. Dentary teeth height near mid-length of tooth row with respect to remaining teeth in posterior half of mandible: equal (0) or enlarged (1).
184. Dentary tooth margin curvature between 3 and 10: linear (0) or gently curved (1).
185. External mandibular fenestra: absent (0) or small and foramen intermandibularis caudalis not visible laterally (1) or large and foramen intermandibularis caudalis visible laterally (2). Ordered.
186. Shape of dentary symphysis in ventral view: tapering, anteriorly forming an angle (0) or U-shaped, smoothly curving anteriorly (1) or lateral edges longitudinally oriented, convex anterolaterally corner, and extensive transversely oriented anterior edge (2).
187. Lateral surface of posterior region of dentary and anterior region of surangular longitudinal depression: absent (0) or present (1).
188. Splenial involvement in mandibular symphysis: absent (0) or present (1).
189. Dentary surface lateral to seventh alveolus: smooth (0) or lateral concavity for the reception of an enlarged maxillary tooth (1).
190. Dorsal edge of dentary orientation to longitudinal axis of skull: slightly concave or straight (0) or straight with an abrupt dorsal expansion anteriorly (1) or single dorsal expansion and concave posterior to this (2) or sinuosoidal, with two concave waves (3).
191. Posterior peg at symphysis: absent (0) or present (1).
192. Dentary compression and ventrolateral surface anterior to mandibular fenestra: compressed and vertical (0) or not compressed and convex (1).
193. Splenial transverse thickness posterior to symphysis: thin (0) or robust dorsally (1).
194. Dentary lateral surface below alveolar margin, at mid- to posterior region of tooth row: vertically oriented, continuous with rest of lateral surface of dentary (0) or flat surface exposed laterodorsally, divided by a ridge from the rest of the lateral surface of the dentaries (1).
195. Angular-surangular contact relative to medial wall of external mandibular fenestra: continue to posterior angle (0) or pass along posteroventral margin (1).
196. Anterior processes of surangular: single (0) or two (1).
197. Coronoid size: short and located below dorsal edge of mandible (0) or anteriorly extended with posterior region elevated at the dorsal margin of mandible (1).
198. Surangular contribution to glenoid fossa: lateral wall only (0) or approximately one -third of fossa (1).
199. Surangular extension toward posterior end of retroarticular process: along entire length (0) or pinched anterior to posterior tip (1).
200. Surangular-articular suture orientation within glenoid fossa: anteroposteriorly (linear) (0) or bowed strongly laterally (1).
201. insertion area for M. pterygoideus posterior on angular: medial (0) or medial and lateral (1).
202. Longitudinal ridge along the dorsolateral surface of surangular: absent (0) or present (1).
203. Sharp ridge on the ventrolateral surface of angular: absent (0) or present (1).
204. Prearticular present (0) or absent (fused to articular) (1).
205. Articular cotyle of lower jaw, shape: wider than long (0) or longer than wide (1).
206. Retroarticular process: short, less than twice the length of the articular cotyle (0) or elongate, equal to or more than twice the length of the articular cotyle (1).
207. Medial edge of retroarticular process: concave or linear (0) or convex (1).
208. Projection of retroarticular process: posteriorly or posteroventrally (0) or posterodorsally (1).
209. Vertebral centra: cylindrical (0) or spool shaped (1).
210. Cervical neural spines: all anteroposteriorly large (0) or only posterior ones rodlike (1) or all spines rodlike (2). Ordered.
211. Axial neural spine height: high, subequal to centrum height (0) or low, less than half centrum height and nearly horizontal (1).
212. Axis neural arch lateral process: absent (0) or  present (1).
213. Postzygapophyses of axis: well developed and curved laterally (0) or poorly developed (1).
214. Anteroposterior development of neural spine in axis: well developed, covering all the neural arch length (0) or poorly developed, located over the posterior half of the neural arch (1).
215. Cervical vertebrae: amphicoelous or amphiplatyan (0) or procoelous (1).
216. Cervical hypapophyses: absent (0) or present only in cervical vertebrae (1) or present in cervicals and at least first two dorsal vertebrae (2). Ordered.
217. Posterior process of cervical rib shaft posterodorsally projecting spine at junction with the tubercular process: absent (0) or present (1).
218. Dorsal vertebrae: amphicoelous or amphiplatyan (0) or procoelous (1).
219. number of sacral vertebrae: two (0) or three or more (1).
220. Caudal vertebrae: all amphicoelous or amphiplatyan (0) or all procoelous (1) or first caudal vertebra gently biconvex and rest procoelous (2). Ordered.
221. Transverse process of sacral vertebrae orientation: lateral (0) or markedly deflected ventrally (1).
222. Scapular blade width relative to length of scapulocoracoid articulation: no more than twice (0) or broad, greater than twice (1).
223. Anterior and posterior margins of scapula in lateral aspect: symmetrically concave in lateral view (0) or anterior edge more strongly concave than posterior edge (1) or dorsally narrow with straight edges (2). Ordered.
224. Deltoid crest of scapula: present (0) or absent (1).
225. Coracoid length relative to scapula: 1/2  (0) or subequal (1).
226. Proximomedial articular surface on humerus: present (strongly arched edge) (0) or absent (weakly arched edge) (1).
227. Longitudinal axis of humeral shaft in lateral aspect: straight (0) or sigmoid (distal end curves anteriorly) (1).
228. M. teres major and M. dorsalis scapulae insertion on humerus: separate, scars distinguished dorsal to deltopectoral crest (0) or insert with common tendon, single insertion scar (1).
229. Olecranon process of ulna: narrow and subangular (0) or wide and rounded (1).
230. Radiale and ulnare length: short (endochondral) (0) or long (perichondral) (1) or long with a distinct proximomedial process on the radiale (2). Ordered.
231. Anterior process of ilium length relative to length of posterior process: similar (0) or one-quarter or less (1).
232. Dorsal margin of iliac blade: rounded with a smooth border (0) or flat (1).
233. Posterior iliac process: dorsoventrally expanded with a blunt end (0) or nearly absent (1).
234. Supra-acetabular crest: present (0) or absent (1).
235. Contribution of pubis to acetabulum: partially excluded by anterior process of ischium (0) or completely excluded from acetabulum (1).
236. Anterior margin of femur: linear (0) or bears flange for coccygeofemoralis musculature (1).
237. Proximal-most portion of fibular head: straight-sided to weakly developed posteriorly (0) or sharply projecting posteriorly, forming distinct extension (1).
238. Fibular articular facet of femur: large (0) or very small (1).
239. Lateral edge of proximal articular surface of femur: rounded (0) or squared with an enlarged ischiotrochantericus muscle scar (1).
240. Fourth trochanter on femur: absent (0) or present but low (1).
241. Tibia length relative to femur length: subequal or longer (0) or shorter (1).
242. Calcaneal facet for fibula and distal tarsal 4: separate (0) or contiguous (1).
243. Calcaneal tuber: absent or rudimentary (0) or 45 degrees posterolaterally (1) or posteriorly (2).
244. Fore and hind limb lengths: hind limb much longer than forelimb (0) or subequal (1).
245. Gap in cervico-thoracic dorsal armor: absent (0) or present (1).
246. Number of dorsal osteoderms per transverse row: none (dorsal osteoderms absent) (0) or two (1) or four or more (2). Ordered.
247. Dorsal osteoderm shape: square (0) or wider than long but less than three times wider than long (1) or more than three times wider than long (2).
248. Anterior edge of dorsal parasagittal osteoderms: straight (0) or discrete convexity on anterior margin (1) or with anterolateral process of anterior edge (2). Ordered.
249. Keel on dorsal osteoderms: absent (0) or present (1).
250. Dorsal trunk osteoderm, anteroposterior keel position: medial or paramedian (0) or lateral margin (1).
251. Ventral trunk osteoderms: absent (0) or present and osteoderms are single (1) or present and osteoderms are paired ossifications sutured together (2). Ordered.
252. Tail osteoderms: absent (0) or dorsal only (1) or completely surrounded (2). Ordered.

**Analysis 3:** Character list from Delfino et al. (2008) [10]. Character numbers and ordering assumptions follow their original.
1. Ventral tubercle of proatlas at least one-half (0) or less than one half (1) the width of the dorsal crest.
2. Proatlas boomerang-shaped (0) strap-shaped (1) or massive and block-shaped (2).
3. Caudal half of axis neural spine wide (0) or narrow (1).
4. Axis neural arch lacks (0) or possesses (1) a lateral process (‘‘diapophysis’’). 5. Atlas intercentrum wedge-shaped in lateral view, with insignificant parapophyseal
processes (0) or plate-shaped in lateral view, with prominent parapophyseal processes at
maturity (1).
6. Axial hypapophysis located toward the center of the vertebral body (0) or toward the
cranial end of vertebral body (1).
7. Hypapophysis present on all cervical vertebrae, along with thoracic vertebrae I and II
(0) or thoracic vertebrae I-III (1) or thoracic vertebrae I-IV (2).
8. First postaxial cervical vertebra without a hypapophysis (0) or with a weakly
developed hypopophysis (1) or with a prominant hypophysis (2).
9. Neural spines on the caudal cervical vertebrae as broad as those on the cranial
cervical vertebrae (0) or neural spines on the caudal cervical vertebrae craniocaudally
narrow compared with those on the cranial cervical vertebrae (1).
10. Proatlas with prominent cranial process (0) or lacks cranial process (1).
11. Cranial half of axis neural spine oriented horizontally (0) or slopes cranially (1).
12. Axis neural spine crested (0) or not crested (1).
13. Cranial sacral capitulum projects far cranially of tuberculum and is broadly visible
in dorsal aspect (0) or cranial margins of tuberculum and capitulum nearly in same
plane, and capitulum largely obscured dorsally (1).
14. Dorsal margin of atlantal rib generally smooth with modest dorsal process (0) or
with prominent process (1).
15. Atlantal ribs lack (0) or possess (1) large articular facets at cranial ends for each
other.
16. Atlantal ribs without (0) or with (1) very thin medial laminae at cranial end.
17. Proatlas has tall dorsal keel (0) or lacks tall dorsal keel (1).
18. Thoracic and lumbar vertebrae amphicoelous (0) or weakly procoelous (1) or
strongly procoelous (2). Ordered.
19. Axial hypapophysis grades into two low ridges caudally (0) or axial hypopophysis
grades into a single ridge caudally (1).
20. Axial rib tuberculum wide, with broad dorsal tip (0) or narrow, with acute dorsal tip (1).
21. Axial rib tuberculum contacts diapophysis late in ontogeny, if at all (0) or early in
ontogeny (1).
22. Scapular blade flares dorsally at maturity (0) or sides of scapular blade subparallel;
minimal dorsal flare at maturity (1).
23. Cranial crest of scapula very thin at maturity, with sharp margin (0) or very wide at
maturity, with broad margin (1).
24. Scapulocoracoid synchondrosis closes very late in ontogeny (0) or relatively early in
ontogeny (1).
25. Scapulocoracoid facet cranial to glenoid fossa uniformly narrow (0) or broad
immediately cranial to glenoid fossa, and tapering cranially (1).
26. Proximal edge of deltopectoral crest emerges smoothly from proximal end of
humerus and is not obviously concave (0) or emerges abruptly from proximal end of
humerus and is obviously concave (1).
27. Proximal extremity of ulna narrow and subangular in outline (0) or wide and
rounded (1).
28. Dorsal margin of iliac blade rounded with smooth border (0) or rounded, with
modest dorsal indentation (1) or rounded, with strong dorsal indentation (‘wasp-
waisted’; 2) or narrow, with dorsal indentation (3) or rounded with smooth border;
caudal tip of blade very deep (4).
29. M. teres major and m. dorsalis scapulae insert separately on humerus; scars can be
distinguished dorsal to the deltopectoral crest (0) or insert with common tendon; single
insertion scar (1).
30. Interclavicle flat along length, without dorsoventral flexure (0) or with moderate
dorsoventral flexure (1) or with severe dorsoventral flexure (2).
31. Cranial end of interclavicle flat (0) or rodlike (1).
32. Supraacetabular crest narrow (0) or broad (1).
33. Limb bones very long and slender; forelimb and hind limb approximately equal in
length at maturity (0) or limb bones relatively robust; hind limb much longer than
forelimb at maturity (1).
34. Cranial process of ilium prominent (0) or virtually absent (1).
35. Dorsal osteoderms keeled (0) or not keeled (1).
36. Biserial dorsal shield, dorsal osteoderms rectangular in outline with distinct medial
and lateral parts either side of a sagittal keel (0); dorsal osteoderms segmented sagittally
into rectangular paravertebral osteoderms and square to round accessory osteoderms (1);
paravertebral osteoderms segmented (2). Ordered.
37. Accessory osteoderms absent (0) or maximum of one longitudinal row of
transversely contiguous accessory osteoderms (1) or maximum of two longitudinal rows
of transversely contiguous accessory osteoderms (2) or maximum of three sagittal
longitudinal rows of transversely contiguous accessory osteoderms (3).
38. Nuchal shield grades continuously into dorsal shield (0) or nuchal shield
differentiated from dorsal shield into four nuchal osteoderms in two parallel rows (1) or
nuchal shield differentiated from dorsal shield into six nuchal osteoderms, with four
central and two lateral (2) or nuchal shield differentiated from dorsal shield into more
than four nuchal osteoderms in two parallel rows (3).
39. Ventral osteoderms present, polygonal (0) or present, square (1) or present, paired
ossifications that suture together (2) or absent (3).
40: Paravertebral osteoderms with a cranial articular surface (0) or smooth, without a
cranial articular surface (1).
41. Splenial with rostral perforation for mandibular ramus of cranial nerve V (0) or
lacks rostral perforation for mandibular ramus of cranial nerve V (1).
42. Mandibular ramus of cranial nerve V exits splenial rostrally only (0) or splenial has
singular perforation for mandibular ramus of cranial nerve V caudally (1) or splenial has
double perforation for mandibular ramus of cranial nerve V caudally (2).
43. Splenial participates in mandibular symphysis; splenial symphysis adjacent to no
more than five dentary alveoli (0) or splenial excluded from the mandibular symphysis;
rostral tip of splenial passes ventral to the Meckelian groove (1) or splenial excluded
from the mandibular symphysis; rostral tip of splenial passes dorsal to the Meckelian
groove (2) or deep splenial symphysis, longer than five dentary alveoli; splenial forms
wide ‘V’ within the mandibular symphysis (3) or deep splenial symphysis, longer than
five dentary alveoli; splenial constricted within the mandibular symphysis and forms
narrow ‘V’ (4).
44. Articular-surangular suture simple (0) or with rostral process dorsal to the lingual
foramen (1) or with rostral process ventral to
lingual foramen (2).
45. Lingual foramen for articular artery and alveolar nerve on surangular entirely (0) or
on surangular/angular suture (1) or on angular entirely (2).
46. Coronoid bounds caudal half of the medial intermandibular foramen (0) or
completely surrounds the medial intermandibular foramen at maturity (1) or obliterates
the medial intermandibular foramen at maturity (2).
47. Angular-surangular suture contacts external mandibular fenestra at caudal angle at
maturity (0) or passes broadly along ventral margin of external mandibular fenestra late
in ontogeny (1).
48. Rostral processes of surangular unequal (0) or subequal to equal (1).
49. Foramen aërum at extreme lingual margin of the retroarticular process (0) or set in
from margin of the retroarticular process (1).
50. Retroarticular process projects caudally (0) or projects caudodorsally (1).
51. Surangular extends to the caudal end of the retroarticular process (0) or is pinched
off rostral to the tip of the retroarticular process (1).
52. Alveoli for dentary teeth 3 and 4 nearly the same size and confluent (0) or fourth
alveolus larger than third, and alveoli are separated (1).
53. Rostral-most dentary teeth directed rostrally (0) or directed rostrodorsally (1).
54. Superior edge of coronoid slopes strongly cranially (0) or almost horizontal (1).
55. Inferior process of coronoid laps strongly over inner surface of Meckelian fossa (0)
or remains largely on medial surface of mandible (1).
56. Coronoid imperforate (0) or with a perforation caudal to the medial intermandibular
foramen (1).
57. Dorsal projection of hyoid cornu flat (0) or rodlike (1).
58. Dorsal projection of hyoid cornu narrow, with parallel sides (0) or flared (1).
59. Process of splenial separates angular and coronoid (0) or no splenial process
between angular and coronoid (1).
60. Sulcus between articular and surangular (0) or articular flush against surangular (1).
61. Surangular with spur bordering the dentary toothrow lingually for at least one
alveolar length (0) or lacking such spur (1).
62. External mandibular fenestra absent (0) or present (1).
63. Dorsal rostral projection of coronoid longer than ventral (0) or ventral projection
longer than dorsal (1).
64. External mandibular fenestra small; caudal intermandibular foramen not visible
laterally (0) or external mandibular fenestra large; caudal intermandibular foramen
visible laterally (1).
65. Surangular-dentary suture intersects external mandibular fenestra rostral to the
rostrodorsal corner (0) or at the caudodorsal corner (1).
66. Angular extends dorsally toward or beyond rostral end of the caudal intermandibular
foramen; rostral tip acute (0) or does not extend dorsally beyond rostral end of the
caudal intermandibular foramen; rostral tip very blunt (1).
67. Surangular-angular suture lingually meets articular at ventral tip (0) or dorsal to
ventral tip (1).
68. Dentary gently curved (0), deeply curved (1), or linear (2) between fourth and tenth
alveoli.
69. Quadratojugal spine prominent at maturity (0) or greatly reduced or absent at
maturity (1).
70. Postorbital bar massive (0) or slender (1).
71. Rostral border of the secondary choanae is comprised of the palatines (0) or
secondary choanae entirely surrounded by pterygoids (1).
72. Secondary choanae project caudoventrally (0) or rostroventrally (1) at maturity.
73. Pterygoidal surface, lateral and rostral to the secondary choanae, flush with choanal
margin (0) or pushed inward to form ‘neck’ (1).
74. Extensive exposure of prootic on external braincase wall (0) or prootic largely
obscured by quadrate and laterosphenoid externally (1).
75. Quadratojugal forms the caudal angle of the infratemporal fenestra (0) or jugal
forms the caudal angle of the infratemporal fenestra (1) or jugoquadratojugal suture lies
at caudal angle of infratemporal fenestra (2).
76. Postorbital contacts neither quadrate nor quadratojugal medially (0) or contacts
quadratojugal, but not quadrate, medially (1) or contacts quadrate and quadratojugal at
dorsal angle of infratemporal fenestra (2) or contacts quadratojugal with significant
descending process (3).
77. Dentary tooth 4 occludes in notch between the premaxilla and the maxilla early in
ontogeny (0) or occludes in a pit between premaxilla and maxilla; no notch early in
ontogeny (1).
78. All dentary teeth occlude lingual to maxillary teeth (0) or occlusion pit between 7th
and 8th maxillary teeth; all other dentary teeth occlude lingually (1) or dentary teeth
occlude in line with the maxillary toothrow (2).
79. Naris projects rostrodorsally (0) or dorsally (1).
80. Quadratojugal extends to superior angle of infratemporal fenestra (0) or does not
extend to superior angle of infratemporal fenestra; quadrate participates in the
infratemporal fenestra (1).
81. Frontoparietal suture deeply within supratemporal foramen; frontal prevents broad
contact between the postorbital and the parietal (0) or suture makes modest entry into
supratemporal foramen at maturity; postorbital and parietal in broad contact (1) or
suture on skull table entirely (2).
82. Supraoccipital exposure on dorsal skull table small (0), absent (1), large (2), or large
such that parietal is excluded from caudal edge of the skull table (3).
83. Quadratojugal sends long rostral process along lower temporal bar (0) or sends
modest process, or none at all, along lower temporal bar (1).
84. Dorsal and ventral rims of squamosal groove for external ear valve musculature
parallel (0) or squamosal groove flares rostrally (1).
85. Palatopterygoidal suture nearly at (0) or far from (1) caudal angle of the suborbital
fenestra.
86. Frontoparietal suture linear (0) or concavoconvex (1).
87. Supratemporal foramen with fossa; dermal bones of skull roof do not overhang rim
at maturity (0) or dermal bones of skull roof overhang rim of the supratemporal foramen
near maturity (1) or supratemporal foramen closes during ontogeny (2).
88. Suborbital fenestra without (0) or with (1) caudal notch.
89. Maxillary alveoli #4 and #5 same size and largest (0) or #5 largest (1) or #4 largest
(2) or homodont (3).
90. Lateral edges of palatines parallel caudally (0) or flare caudally, producing a ‘shelf’
(1).
91. Ectopterygoid abuts maxillary toothrow (0) or maxilla broadly separates
ectopterygoid from maxillary toothrow (1).
92. Shallow fossa at rostromedial corner of supratemporal foramen (0) or no such fossa;
rostromedial corner of supratemporal foramen smooth (1).
93. Lacrymal makes broad contact with nasal; no caudal process of the maxilla (0) or
maxilla sends caudal process within the lacrymal (1) or maxilla sends caudal process
between the lacrymal and the prefrontal (2).
94. Lateral edges of palatines smooth rostrally (0) or with lateral process projecting
from palatines into suborbital fenestrae (1).
95. Naris bisected by the nasals (0) or nasals contact the naris, but do not bisect it (1) or
nasals excluded, at least externally, from the naris; nasals and premaxillae still in
contact (2) or nasals and premaxillae not in contact (3).
96. Palpebral forms from single ossification (0) or from multiple ossifications (1).
97. Premaxilla has five teeth (0) or four teeth (1) early in posthatching ontogeny.
98. Caudomedial processes of pterygoid prominent and project ventrally (0) or small
and project caudoventrally (1) or small and project caudally (2).
99. Prefrontal pillar solid (0) or with large pneumatic sinus (prefrontal recess) (1).
100. Prefrontals separated by frontals and nasals (0) or prefrontals meet medially (1).
101. Dorsal surface of rostrum curves smoothly (0) or bears medial dorsal boss (1).
102. Caudal margin of otic aperture smooth and continuous with the paraoccipital
process (0) or caudal margin of otic aperture inset (1).
103. Margin of orbit flush with skull surface (0) or dorsal edges of orbits upturned (1) or
orbital margin forms a distinct collar (2).
104. Medial parietal wall of supratemporal foramen imperforate (0) or bearing smaller
foramina (1).
105. Lateral edge of suborbital fenestra straight (0) or bowed medially (1).
106. Surangular continues to dorsal tip of lateral wall of glenoid fossa (0) or truncated
and not continuing dorsally (1).
107. Caudal rim of secondary choanae not deeply notched (0) or deeply notched (1).
108. Rostral margin of palatine process rounded or pointed rostrally (0) or rostrally
concave (1).
109. Rostral ectopterygoid process tapers to a point (0) or forked (1).
110. Palatine process extends (0) or does not extend (1) significantly beyond the rostral
end of the suborbital fenestra.
111. Maxillary foramen for palatine ramus of CN-V small or not present (0) or very
large (1).
112. Quadrate with small, ventrally-reflected medial hemicondyle (0) or with a small
medial hemicondyle; dorsal notch for foramen aërum (1) or with a prominent dorsal
projection between the hemicondyles (2) or with an expanded medial hemicondyle (3).
113. Basisphenoid expanded rostrocaudally ventral to the basioccipital (0) or forms a
thin sheet (1).
114. Quadratojugal spine low, near caudal angle of infratemporal fenestra (0) or high,
between caudal and superior angles of infratemporal fenestra (1).
115. Laterosphenoid bridge comprised entirely of laterosphenoid (0) or includes an
ascending process of the palatine (1).
116. Pterygoidoectopterygoidal flexure disappears during ontogeny (0) or remains
throughout ontogeny (1).
117. Lacrymal longer than prefrontal (0), or prefrontal longer than lacrymal (1), or
lacrymal and prefrontal both elongate and nearly the same length (2).
118. Palatine process generally broad rostrally (0) or in the form of thin wedge (1).
119. Basisphenoid not broadly exposed ventral to the basioccipital at maturity in
occipital aspect; pterygoid dorsoventrally short ventral to median eustachian opening (0)
or basisphenoid broadly exposed ventral to the basioccipital in occipital aspect at
maturity; pterygoid dorsoventrally tall ventral to median eustachian opening (1).
120. Medial jugal foramen small (0) or very large (1).
121. Quadrate foramen aërum on mediodorsal angle (0) or on dorsal surface (1) of
quadrate.
122. Sulcus on rostral braincase wall lateral to the basisphenoid rostrum (0) or braincase
wall lateral to the basisphenoid rostrum smooth; no sulcus (1).
123. Skull table surface slopes ventrally from median plane (0) or planar (1) at maturity.
124. Incisive foramen absent or small, less than half the greatest width of premaxillae
(0) or large, more than half the greatest width of premaxillae (1) or large, and intersects
maxillopremaxillary suture (2).
125. Vomer entirely obscured by premaxilla and maxilla (0) or exposed on palate at the
maxillopremaxillary suture (1).
126. Vomer entirely obscured by the maxillae and the palatines (0) or exposed on palate
between the palatines (1).
127. Significant ventral quadrate process on lateral braincase wall (0) or
quadratopterygoidal suture linear from basisphenoid exposure to the foramen ovale (1).
128. Lateral carotid foramen opens lateral (0) or dorsal (1) to basisphenoid lateral
exposure at maturity.
129. Basisphenoid not exposed extensively (0) or exposed extensively (1) on braincase
wall rostral to foramen ovale.
130. Capitate process of laterosphenoid oriented laterally (0) or rostrocaudally (1)
toward the midline.
131. Parietal and squamosal widely separated by the quadrate on the caudal wall of the
supratemporal foramen (0) or the parietal and the squamosal approach each other on
caudal wall of supratemporal foramen without actually making contact (1) or the
parietal and the squamosal meet along caudal wall of the supratemporal foramen (2).
132. Quadratosquamosal suture extends dorsally along caudal margin of the external
auditory meatus (0) or extends only to the caudoventral corner of the external auditory
meatus (1).
133. Ectopterygoid extends along the medial face of postorbital bar (0) or stops abruptly
ventral to the postorbital bar (1).
134. Rostral projection on postorbital bar prominent (0) or not prominent/absent (1).
135. Maxillary tooth row laterally convex or parallel (0) or laterally convex and flaring
caudally (1) caudal to first six maxillary alveoli.
136. Medial process of prefrontal pillar expanded dorsoventrally (0) or rostrocaudally
(1).
137. Dorsal half of prefrontal pillar narrow (0) or expanded rostrocaudally in dorsal half
(1).
138. Medial process of prefrontal pillar wide (0) or constricted (1) at base.
139. Ventral margin of orbit gently circular (0) or with prominent notch (1).
140. Mature skull table with broad lateral curvature; short caudolateral process of the
squamosal (0); mature skull table with nearly horizontal sides; significant caudolateral
process of the squamosal (1); mature skull table with nearly horizontal sides;
caudolateral process of the squamosal greatly enlarged (2).
141. Exoccipital with prominent boss medial to the paroccipital process; process lateral
to the caudal aperture of the cranioquadrate canal short (0) or exoccipital with small or
no boss medial to the paroccipital process; process lateral to the caudal aperture of the
cranioquadrate canal long (1).
142. Premaxillary surface lateral to the naris smooth (0) or with deep notch (1).
143. Rostral canthi absent or very modest (0) or very prominent (1) at maturity.
144. Preorbital ridges absent or very modest (0) or very prominent (1) at maturity.
145. Dorsal premaxillary processes short, not extending beyond third maxillary alveolus
(0) or long, extending beyond third maxillary alveolus (1).
146. Dorsal process of the jugal flush with the lateral surface of the jugal (0) or
considerably inset from the lateral surface of the jugal, and displaced ventral to the
dorsal margin (1).
147. Lateral eustachian canals open dorsal (0) or lateral (1) to medial eustachian canal.
148. Surface of the maxilla within the narial canal imperforate (0) or with multiple cecal
recesses (1).
149. Ectopterygoid extends (0) or does not extend (1) to caudal tip of the lateral
pterygoid flange at maturity.
150. Squamosal does not extend (0) or extends (1) ventrolaterally to lateral extent of
exoccipital and quadrate.
151. Exoccipital terminates dorsal to basioccipital tuberosity (0) or sends a robust
process ventrally and participates in the basioccipital tuberosity (1) or sends a slender
process ventrally to the basioccipital tuberosity (2).
152. Secondary choanae not septate (0) or with a septum that remains recessed within
the choanae (1) or with a septum that projects out of the choanae (2).
153. Incisive foramen completely situated far from the premaxillary toothrow, at the
level of the second or third alveolus (0) or abuts the premaxillary toothrow (1) or
projects between first premaxillary teeth (2).
154. Parietal with sinus communicating with a pneumatic system (0) or solid, without a
sinus (1).
155. Ventral scales have (0) or lack (1) follicle gland pores.
156. Ventral collar scales not enlarged relative to other ventral scales (0) or in a single
enlarged row (1) or in two enlarged parallel rows (2).
157. Median pelvic keel scales form two parallel rows along most of tail length (0) or
form single row along tail (1) or merge with lateral keel scales to form Y-shaped keel
(2).
158. Lingual osmoregulatory pores small (0) or large (1).
159. Tongue with (0) or without (1) keratinised surface.
160. M. caudofemoralis with a single head (0) or with double head (m. caudofemoralis
longus and m. caudofemoralis brevis; 1).
161. Naris circular or keyhole-shaped (0) or wider than long (1).
162. Suranguloarticular suture oriented rostrocaudally (0) or bowed strongly laterally
(1) within glenoid fossa.
163. Postorbitosquamosal suture oriented ventrally (0) or passes medially (1) ventral to
skull table.
164. Rostral foramen for palatine ramus of cranial nerve VII ventrolateral (0) or ventral
(1) to basisphenoid rostrum.
165. Caudal maxillary alveoli round (0) or mediolaterally compressed (1).
166. Dentary symphysis extends to sixth through eighth dentary alveolus (0) or to fourth
or fifth alveolus (1) or beyond eighth dentary alveolus (2).
167. Largest dentary alveolus immediately caudal to fourth is: 13 or 14 (0); 13 or 14 and
a series behind it (1); 10, 11, or 12 (2); or, all alveoli are of a uniform size (3).
168. Prefrontal pillars transversely expanded at their dorsal half and columnar ventrally
(0); narrow or longitudinally expanded at their dorsal part and columnar ventrally (1).
169. Height of peduncle of neural arch on caudal cervical vertebrae approximately
equivalent to that of peduncle on neural arch of each of the thoracic, sacral and cranial-
most caudal vertebrae (0) or considerably greater (1).
170. Cervical vertebrae all amphicoelous (0) or some amphicoelous and some
procoelous (1) or all procoelous (2). Ordered.
171. Caudal vertebrae all amphicoelous (0) or first caudal vertebra opisthoceolous or
procoelous, remainder of caudal vertebrae amphicoelous (1) or first caudal vertebra
opisthoceolous or procoelous, remainder of caudal vertebrae procoelous, with the
degree of procoely decreasing terminally (2) or first caudal vertebra biconvex,
remainder of caudal vertebrae procoelous, with the degree of procoely decreasing
terminally (3). Ordered.
172. Secondary choanae situated near the rostral margin of the pterygoids (0) or towards the caudal margin of the pterygoids (1).
173. Distal extremity of the ulna expanded transversely with respect to the long axis of
the bone; maximum width equivalent to that of the proximal extremity (0) or proximal
extremity of the ulna considerably wider than the distal extermity (1).
174. Occipital surface ventral to basioccipital condyle slopes rostroventrally (0) or is roughly parallel to the transverse plane (1).
175. Postorbital bar continuous with the lateral edge of the dorsal part of the postorbital (0) or postorbital bar inset from the lateral edge of the dorsal part of the postorbital (1).
176. Antorbital fenestra present (0) or absent (1).

**Matrices used in phylogenetic analysis. Nexus files can be requested from CM Holliday (hollidayca(at)missouri.edu)**

SERENO AND LARSSON (2009) MATRIX

*Orthosuchus stormbergi*

2101000400 1?00000{12}00 2?10001000 20000010?1 0000?00202 100111020? 0000000000 01101?0110 00020-200- 0010000?01 -0200000-0 01000?00?0 0---?00??0 00??00?0?0 ?10??00011 1000000002 101000-00? 100??00?30 01012?0000 ???000?01? 00000????0 001000?000 ?010000??2 000000?101 0121011211 02

*Zosuchus davidsoni*

20011?0{34}?0 {01}0???00??? {12}??0?1000? 2???001??1 010000?2?? 0?{01}11??211 000?10??{123}? 0?11??0??{01} ???0??1?1? 0?00000??1 ?02?01?0?0 011000000? 0???0?010? ??00010??0 11?00????? 01{01}10????2 1?10????2? 0000?00000 0?1??00000 ?100???0?? ?00?0????? ?????????? ?????????? ?????????? ?????????? ??

*Hsisosuchus chungkingensis*

2101100400 0000?00200 0000000000 2010001010 0110000201 1?11120200 0000100010 00100?0111 000012301? ?00??0??01 -??00111-? 00?10121?? 0---001100 0000000000 01000?001? {01}??10?1101 100000-001 000??000?? 1??01?01?? 00?1?0?00? 001?0010?? ????00?0?0 ?0100?1??2 ???????1?? ???1011010 12

*Pelagosaurus typus*

2001201000 1110000000 0000011000 0010000001 0010000100 11000000-0 1001000000 0000211000 10001?2?1? 0000000000 ?0011-0001 000000-001 1101101000 000000{01}101 010?000001 1000001100 0110000000 110??01000 ??--21110- 0??01??0?? 00000101?0 01??00?0?0 ?01?1????1 010100?1?0 11{12}001120- 11

*Steneosaurus bollensis*

1001201000 1110000000 0000011000 002-000001 0110?0?000 11000000-0 1011100000 00002?1000 200011211- 0000000000 000?1-000{01} 000000-00? 1000101001 00000001?1 010?001000 100000110? 0110?1000? 110??110?0 00--21010- 0100?01001 0000010100 101000?000 1?11100??1 010100?100 1110011210 11

*Metriorhynchus superciliosus*

0001211000 1110110001 0010011000 0010000000 0?1000?000 11000000-0 1111100000 00002?0001 100001101- 0000000000 000-1-0001 000000-00? 2101101000 000000?101 010?0000-0 1000001100 011001000? 110??110?0 -0--0?110- 0?00-0100? 0000000100 11?000?000 1?01110??0 001100?000 1010-0---- 00

*Geosaurus suevicus*

0001311000 111011000? 0020011000 002-00010? 0010????00 11000000-? 111?100000 00002??000 10?0??101- ?00?000000 ?00?1-000? 000000-0?? ?????????? ?????????? ?????????? ????0??10? 0??0???00? ?????010?0 ?0--00110- ???0-0???? 000?00?1?? 11100??0?0 ??01110??0 ?01?00?000 1000?0---- 00

*Uruguaysuchus aznarezi*

20011?0{34}?0 {01}???0?0??? ?????1??0? 2???011??1 ?1?0?0??0? ???11??{01}?0 ??0??????? ?????????? 2??21?2?1? ??0???{02}??0 ??011100?? 10?2010{01}1? 1????????? ???0?0?0?? ?{12}?0?0???? ???111{12}??1 ???1?????? 0????00001 ?001{12}?0100 1????????? 1???1???0? ???????0?0 ?????????2 1??0111??? ??????001? ??

*Notosuchus terrestris*

1011100200 1000100001 1120010101 2011011001 0110001100 00?1110100 000112?010 0110110120 2002123?1- 0000000001 -0001010?0 1112110001 110{01}101110 00100000?1 1100111101 0111111212 1101?10121 0000100010 100020{01}100 10010??01? 100?100002 ??0100?010 011?10???? 1??0110??? ?????1101? ??

*Malawisuchus mwakasyngutiensis*

1011100{34}00 0000000{12}01 0?10?10{01}01 20010110-1 0000??1100 0?011?0100 ?00112??10 0?10{01}??1{12}2 20?2??2010 ?00?00{02}001 ?0101000?? 1112001011 110{01}10100? 00000000?1 ?1000?110? 011101?211 1101?1012? 00?0?0001? 1?00200100 00?100?01? 100?1010?2 ????01?0?0 ?01??00??? ?????1?1?? 0?11?20010 ??

*Mariliasuchus amarali*

10113-0{34}?0 ????100??1 0??0110{01}?? 2????1101- 00101001?? 0?0111?110 0001120030 0111110??2 20?01?2?1- 0000100001 ?000101000 111001111? 1-12101111 01001000?1 0100112000 00011112?2 1101??0??? 0010?00??0 1000200100 10?1?1?010 10011010?? ?????????? ?????????? ?????????? ?????????? ??

*Comahuesuchus brachybuccalis*

10123-0{34}?0 {01}0??100??? ???011010? ?????????- 00??1???0? ????1????0 ?00??????? ?????10??2 2?????{23}?1? 2?0?100000 ??0011?0?0 110?0?10?1 1???1??01? ??1??????? ????101?0? 0?1111???1 ??0??1??2? ????1??00? ?0???10102 10?1?????? ??0??????? ?????????? ?????????? ?????????? ?????????? ??

*Sphagesaurus huenei*

10113-?{34}?0 ?0????0??? ????0????? ????01101- 101000110? 0?1??????? ???11200?0 ?????101?2 2002000?1? 0?00000001 0000101000 110011-011 1??11?10?? ???00010?1 ?200?????? 01?11????? ??0??????? ?110??0010 ???0?????0 ???1???0?? ?????????? ??01?????? ?????????? ?????????? ?????????? ??

*Baurusuchus pachecoi*

1000{13}?0310 ??00?00?0? 0110011?01 ?01001111? 10110?1101 0{01}010110-0 00?1121010 0110110020 200{01}1?2010 0000000001 ?021111000 1101011111 210210100{01} {01}010{12}010?1 110?1111?? 01111112?1 1101??0121 0000?00120 11012{01}1102 00100?001? 110100000? ???????010 0???101??? ?000?1???? 0?{12}1??0??? ??

*Baurusuchus salgadoensis*

1000100310 0000100{12}0? 0100011001 2010011111 111100?101 0{01}010110-0 0001121010 0110???022 200{01}1?2010 ??0?000001 ?021111000 11?00011?? 210210100{01} ?0?0{12}1?0?1 0???1?1?0? 01??11{12}2?? 1101????2? 00???00120 1101211102 00?00???1? 110?0010?? ?????????? ?????????? ?????????? ?????????? ??

*Anatosuchus minor*

2002100400 0000000202 0?10010100 ?100211101 0011000100 1211120110 0001121030 01101?0122 210?1{12}3010 ?000001010 ?000110000 10?00?001? 1???1?1000 {01}0002??0?? 0?1000???? 01?12122?1 1101?1?11? 0?0??00020 ?001220100 00000???0? 1011?100?? ????????0? 0110100??2 ?????????? ????011011 ??

*Simosuchus clarki*

2012100400 0000000{12}0{12} 0?10010100 2210201100 0011000101 1211120110 0001122030 01100?0032 2102113010 2000001010 0000111000 1012010010 0-?2101000 00002010?{01} 1210001?0? 0110211201 1100?1011? 0000?00020 1001220100 100001?000 10111010?2 ????01?0?? ?????????? ?????????? ?????2001? 1?

*Araripesuchus gomesii*

2002100300 0000000201 0010010001 2000211011 00100?0101 1101110??0 0001120031 0110110120 211212201- 000?002000 -001111000 1011001111 1102101001 ?0001000?1 0211001101 01010112?1 1?11?1?111 0000100001 10?12{01}0101 00?00??11? 10010110?? ??00021010 011?????02 1??01111?? ????011011 02

*Araripesuchus buitreraensis*

{12}002??0??0 10??0????? ?????10?0? 2????11??? ?010???10? ???11????? ??01122??? 0?10?????? ????????1? 0?0?0????? ??01110??? 1?100010?1 1??11?1002 ??0020?0?1 121100{123}??? ?????????1 ??????1??? ????1??0{01}? ?1?1?011?{01} 100001???? ?????????1 ?????????? ?????????? ?????????? ?????????? ??

*Araripesuchus tsangatsangana*

2002100{34}00 0000000?0{12} 2??001000? 2001?11??1 00100?110? 0?011?0??0 000112{12}??1 0?10100??0 2?121?3?1? ??0?{01}020?1 ?0011110?0 1010011011 1???1?100? ?0??100011 120000{123}??? 01{01}111?2?1 1?10?10??1 00?0100001 10112001?1 10000???1? 101111101? ???0021000 ?1101000?2 1??00111?? ??????10?? ??

*Araripesuchus patagonicus*

2002100{34}00 0000000?0? 0??0?1000? ?0002{01}1??1 00100?0100 0?011?0110 0001121?3? 01101?0?2? 2??{12}??2?1? ??0?0??0?1 ?0011100?0 1010001??? 1???1?1?0? ??001000?1 021100{123}?0? 010?11?2?1 ??01?10?1? 000??0000? 1???20010? 1?0?0???1? 100?1110?1 ??????1??? 011???0??? 1??0??1??? ????011011 ??

*Araripesuchus wegeneri*

2002100{34}00 0000000201 0??001010? ?001211111 0010000100 1211120210 0001121?31 0110110122 21120?3?1? 0?000020?1 00011100?0 1012011111 1??11?1002 2000200011 0211003?0? 0111112211 1?01?11?01 0000100001 ?111?0???0 ?0?0?????? ?????????? ?????????? ???010???? ?????????? ???1????1? ?2

*Araripesuchus rattoides*

20???????? ?????????? ?????????? ?????????? ?????????? ?????????? ?????????? ?????????? ?????????? ?????????? ?????????? ?????????? ?????????? ?????????? ?????????? ?????????? ?????????? ???????001 ?111?0?10{123} ?0?0?????? ?????????? ?????????? ?????????? ?????????? ?????????? ??

*Sebecus icaeorhinus*

10002?0{34}10 ?000?0?20{01} 0010?10101 ?{12}11011011 01110001?0 201112021? ??0112???? ????1?0?{12}? 21?1002011 0100000001 10211200?0 100101111? 110010100{01} {12}000{12}0???1 ??????1110 1001012211 1100?11101 010???001? ?111?{01}0002 ???0?1?0?0 ??010?11?? ????0??0?? ?????????? ???????11? ?????????? ??

*Hamadasuchus rebouli*

2002200400 100000020{12} 0010010101 ?200011011 0011000110 2111120200 0001122031 0111100122 2101012011 0100000001 0021120010 1011011110 2002101002 2000200011 020?002110 1000012201 1100111101 0100110010 ?111?0?102 00???????? ?????????? ?????????? ?????????? ?????????? ?????????? ??

*Stolokrosuchus lapparenti*

2001100400 0110000201 0000010011 ?111210011 0011000110 2211120210 0001121031 0110110121 2101003121 0100000001 1121121010 1011012011 {12}??01?1002 2?0{01}200011 ?{12}????11?1 1001012210 110011111? 0101100010 010020010- 01100??0?? 100101?1?? ?????????? ?????????? ?????????? ?????????? ??

*Uberabasuchus terrificus*

2002000300 0?00000{12}0? 2010010001 2100011011 0{01}11000{01}00 2111120210 000112?01? 01101?0121 210???3111 ??0??00001 ?1211200?? 10?1011011 {12}??????0?? ?????????? ?????????? ????01{12}2?1 1100????0? ???????0{12}0 11?1100112 001??1??1? 00010001?? ????0??0?0 ?????????? ?????????? ?????1???? ??

*Peirosaurus tormini*

20022?0?0? ?????0??0? 2?{12}0?100?1 ??0??????? ?????????? ???11????0 ?00??????? ???????12? 210?00311? 0?0?000001 112112???? 10??0?10?1 {12}????????? ?????????? ?????????? ?????????? ???0??1??? ??????0??? ?1?1{12}??1?? ?????????1 ??????0??? ?????????? ?????????? ?????????? ??????0?10 ??

*Lomasuchus palebrosus*

20022?0?00 001000020{12} 2010?10001 22012100?1 00110?01?0 211112?200 ?00112{12}0?? 0110110??? ???100??1? {01}?0??0?000 ?121120010 1011011011 {12}??21?1002 2?00200011 02000?1??? ???????2?1 11?0?1110? 01001000?0 ?11?{12}?0??? ?0?0?????? ?0???????? ?????????? ?????????? ?????????? ?????????? ??

*Mahajangasuchus insignis*

2002100{234}01 000?0002?1 201001001? ?111210??1 0012010101 1{01}11121310 0001122031 00102100?1 10011{12}{23}?1? 2?000000?0 0021121000 100001100? 2102111000 11012000?1 021000211? 100001{12}200 1101?10?21 0100?00120 1111110012 00101??1?1 1101010011 ????0210?0 ?110?01??2 1000110111 1121?10110 ??

*Kaprosuchus saharicus*

2002100401 0000000201 201001001? ?111210??1 0012010{01}01 111112?310 000112??31 0010{12}10011 100{01}1{12}101? ?1000110?1 0021121000 10?001100? 2--2111000 11012000?1 021000{23}110 100001?20? 1?01????01 0?0???0120 0?1?110002 001001?1?? 11110100?0 ?????????? ?????????? 1????????? ?????????? ??

*Goniopholis simus*

20023-0100 101000011{12} 00110100-0 22013110?- 0011000120 1?11121211 000112?021 10101?0001 100{01}113010 ?000000000 0021120100 10?0002001 1102101001 1?0020?0?1 020?0021?? 1000012210 1100???10? 010??00030 ?111?1?{12}03 ?110???0?? 000100?01? ??100??0?0 ?01??????{12} ???1?0?1?? ????011201 {12}?

*Pholidosaurus purbeckensis*

21013-0000 101100000? 0001?110?0 ?20121000- 0?1??00120 11?1121111 000111??{12}1 10101????? ????1???2? ??0??????? ?0{01}?1-000? 10?000?00? 1?0?111001 010120?0?1 ?{12}0?00110? 100001{12}200 1100?1?100 0????0???? ???-???10- ??0??????? ????0????0 ???????0?? ????1????? 1???10???? ?????12211 1?

*Dyrosaurus phosphaticus*

10013-1101 101000?001 0001?10010 122-11100- 0210??01-0 2?01121211 001?112?21 10101?0011 1000112020 ?00?000000 ??201-000? 100000?00? 2110111001 010120???1 ?20010211? 100?012200 1100011100 01??1110?? 1?--???10- ??00???1?? ?00?01?0?? 11??0??0?0 ????10???? ?00?10??11 ?????12?0- ??

*Terminonaris robusta*

20013-1100 101?00000? 00010110-0 ?21?{23}1000- 0?1??00120 1?1112?201 000??1??{12}1 10101??000 1000013020 ?011011110 00201-0000 10?000?0?1 11001{01}1000 ?10{01}20?0?1 0{12}0000?10? ?0000??20? ??00?1?10? 0?0?1100?? 1?--1101?- 0??1???00? 1??1010?10 1?10000000 ?010100??2 1101100111 1111?12211 2?

*Sarcosuchusimperator*

20023-0100 {01}011001001 0001011010 ?20011000- 0111000110 1111121111 0001110121 10101?0000 100011302- 2011011111 001{01}1-0000 1010002001 1001111001 1101200011 02?000110? 1000012200 110001?10? 01???100?0 10--11010- 0110110011 000101101? ????000000 0?10?????? 11011????? ?????12211 1{12}

*Bernissartia fagesii*

20023-0{23}00 101000000{12} 1111?10010 ?{12}003110?- 0{01}11?00121 ?11112???1 0?011{12}1??1 00101??0{01}{01} 200???301? ??0?000000 ?01112?0?0 10100120?? 200{01}11100{12} ?101{12}1?0?? 0?????211? 10??0122?? 1100????1? ?1?????000 -111010003 01?0-??01? 001100011? 1??011?000 ?????0???? ?????????? ????12{01}010 1?

*Isisfordia duncani*

20023-0300 ?01000000? 0000010010 ?20131111- 0011000{01}21 1?11121111 000112??{12}? 0010???010 1000113010 1000000000 0101100000 1010010011 2111121001 01022000?1 0200002?0? 100001{12}20? 1100?1011? 0100?00??? 000?{12}?0??? ???0?1???? ????????1{01} 1?101{12}?101 ?01??01??2 100???0?1? 1?{12}1120010 1{12}

*Gavialis gangeticus*

21013-0000 1011001001 0001011011 100011001- 0010000120 0111121101 000112?111 0010100000 1000113010 2000000000 00011-0000 100000-001 2100131001 1102210011 00--002111 1000012200 1?0??0??11 0101011030 01--11010- 0100110010 1001010111 1?10120102 0010101012 1001100111 11??020110 02

*Leidyosuchus canadensis*

20023-0300 1010000102 0010010001 120021100- 0011000110 0111121201 0001120121 0010100011 1001103010 1000000000 0021120000 1010012011 2001132001 1102211011 00--00310? 1000012201 1100?00101 0101110030 0111110103 ?1?0010?10 10010101?? ????1??1?2 ????1??1?2 ?0??1??1?? 1??0?20010 1?

*Crocodylus niloticus*

20023-0300 1010000102 0000010011 121031100- 0111000122 0011121101 0001120121 0010100010 1000113010 2000000000 0021120000 1000002011 1101131001 1102210011 00--002100 1000012201 1100100101 0101110030 0111110003 0100110001 1001010111 0010120102 0020101112 1001100111 1120120010 02

*Alligator mississippiensis*

20023-0400 1010000212 2120010011 120121001- 0111000110 0111121101 0001121121 0010100010 1001113010 1000000000 1011120000 1010012011 2012132002 2102210011 0200003100 1000012201 1100100101 0101110030 0111210003 0100110010 1001011111 0010120102 0020101112 1001100111 1120120010 01

*Laganosuchus thaumastos*

20???????? ?????????? ?????????? ?????????? ?????????? ?????????? ?????????? ?????????? ?????????? ?????????? ?????????? ??00???00? ?????????? ?????????? ?????????? ?????????? ?????????? ???????030 1100111000 0100?-0010 11010110?? ?????????? ?????????? ?????????? ?????????? ??

*Aegyptosuchus peyeri*

2?????0??1 001000020? ???0?10201 {12}200?????? ?????????? ???1111201 0100?2??1? 11?0?????? ?????????? ?????????? ?????????? ?????????? ?????????? ?????????? ?????????? ???????2?? 11???1??11 01??0????? ?????????? ?????????? ?????????? ?????????? ?????????? ?????????? ?????????? ??

*Aegisuchus witmerii (ROM54530)*

2?????0??? ?????????? ?????10201 {12}200?????? ???????1?0 ???1111??1 0100?22?11 11?0?????? ?????????? ?????????? ?????????? ?????????? ?????????? ??????1?0? ?????????? 1????122?? 110?010111 010101???? ?????????? ?????????? ?????????? ?????????? ?????????? ?????????? ?????????? ??

**DELFINO ET AL. (2008) MATRIX**

*Theriosuchus spp.*

?000?1?11? 000????000 ?000000000 0000000010 0000??00?0 111?????01 10?0000011 0000021000 0310000000 0010000000 0110000000 000?001000 1010000000 01000?0?00 ?000010??0 01???????? 00??002001 100000

*Goniopholis spp.*

?000?0?00? 000???00?? ?0000000?0 0000000000 00000?0000 001??????1 11?0000000 000?000010 0100000000 0001200000 0000000000 0000?00000 0?10000??0 ?000000000 000000?0?0 ?1???????? 00??000?00 001001

*Bernissartia fagesii*

??11?1211? 010????00? ?0?0?0000? ?000011010 0000????00 100???00?1 10???0?000 0???010000 00?0100000 1?101?00?0 0100100?00 ?010?000?0 0??0?????? 0??00???00 0000010?00 001??????? 00??002000 30?011

*Susisuchus anatoceps*

????????1? ??01???0?0 ?000?00??? ??0?021310 ?????????1 1????????1 ????????10 ????011010 02?0?00?3? ?12?2?0??0 0?0000???? 00????0??0 0?1??????? 2??00???00 100000???0 ?????????? 0???0???10 0?0011

*Hylaeochampsa vectiana*

?????????? ?????????? ?????????? ?????????? ?????????? ?????????? ?????????0 100??1?0?0 00?01000?0 0110???0?0 00200?0000 ?01??0101? ??0??000?1 0000?0?000 0?000000?0 00???????? ??0?0??0?? ?1?111

*Isisfordia duncani*

?00?01?101 100???11?? ?000??01?? ?000021110 ?????????? ?????????? ?1?????00? 100?0??0?? 00?0??0??0 01?1???00? ?1?00?0000 ?000???00? 0?1??????? 010??????0 1????10?10 01???????? ??0?0?3?02 200011

*Borealosuchus formidabilis*

0000010110 1100??02?0 0000000010 0000120?20 0000000?01 0010?00100 1100000001 100?0?0200 0000100000 0100200000 0100000000 0010?02110 0?100000?? 010?101001 1000010?10 001??????? 000?003112 310111

*Borealosuchus wilsoni*

???????11? ??0????2?? ?00000001? ??00120?20 ??10200001 0010?0???0 11?0000001 1000010200 100?100000 01002?0000 0100000?00 ?01000211? ?01????0?? 01001?1?01 1????1??10 001??????? 000?012?12 311111

*Borealosuchus acutidentatus*

?????????? ?????????? ??????0??? ?????????? ?????????? ?????????? ???0000001 ????0?020? 1000?00?0? 01002?00?0 01000???0? ?0????2??? ??10???0?? ????1???01 1????1???0 001??????? 000?01???? ??1111

*Borealosuchus sternbergii*

000001?110 1100001210 0000000010 000012???0 0000000001 00100000?0 0100000001 1000010100 0000110100 01012?0000 0100000000 0010?00011 0010000001 01011?1?01 1000010010 0010?????? 000?002?12 311111

*Leidyosuchus canadensis*

????01???? ??0????2?? ?00001?01? ??1102??11 0000000111 1010?0???1 ?100000001 1000010000 0000110100 11011?0000 0200100000 0111000010 1010000001 0101001001 1000010010 0010?????? 0001000??? 31?111

*Eothoracosaurus mississippiensis*

?????????? ???????2?? ??000????? ?01?12???0 ??30???001 1?1??????0 ???????2?0 100?01021? 00?1110030 01?02?00?0 0100000000 001???0100 0?0?0????? ?1?00???01 1000100??? 00???????? 00??023??2 31?1?1

*Thecachampsoides minor*

???1?1???? 0??????21? ?00?0?100? ?010120??0 ?030000001 1110?0??00 ?1?00?02?0 100?010210 1001110030 01002?0000 0?10000000 030???0100 000000?0?1 0100?0?001 1000100?1? 100??????? 000?023??2 ?1??11

*Siquisiquesuchus venezuelensis*

?????????? ?????????? ??1??????? ??1??????? ??30?0??01 ?110?0???? ???????2?0 1???0?021? ?0?1??0?30 01?0??02?? 01200??000 ?00????10? 0?0????0?? ???0????12 1000100??? 1????????? 00??023??? ????11

*Thoracosaurus macrorhynchus*

??11?1?1?? 0??????21? ?????0??0? ???012???0 ??300??001 11???????0 ?100000200 100?010210 0001100030 01002??000 0110000000 0010?1010? 000000?0?0 ?1000?0?01 1000100?10 000??????? 000?023??? 31??11

*Eogavialis africanum*

?????1???? ??0?????11 ?????????? ???0?????0 ?030??10?1 111??????1 ?1?0000200 1000010210 1101110030 01002?0000 0120000000 0000000100 000?00?0?0 0100?00011 1000100?10 1000?????? 000?023??? ?1??11

*Gryposuchus colombianus*

??1?0????? 00?????2?? ??000????? ?????????0 ?030000001 111100???0 0100000200 1001010210 1001100030 01102?0200 0120000000 ?000000100 0000000000 0100????12 1000100?10 100??????? 000?023??? 31?011

*Gavialis gangeticus*

0211010100 0000000210 0000001001 00100?0030 0030000001 1110000100 0100000200 1000010210 1000100030 0110300200 0120000000 0000000100 0000000000 0100?00011 1000100010 1000000000 0000023102 311011

*Pristichampsus spp.*

??000101?? 01?????20? ?00000111? ?01002???0 00001?0??1 ?11??????? ?1?00000?1 100?010000 2000110000 01001?0000 0110000000 021?000010 0?1000?1?1 02010???01 1000010?10 001??????? 010?102?02 311111

Diplocynodon darwinii

100000?210 0101000201 ?000?1141? ?011021120 0010100111 101??000?1 ?1?0000011 1?0?011000 0000100101 ?1002?0000 0200000000 ?111?00010 1?1000?0?? 12?10???01 1000010?10 001??????? 000?000112 311111

*Baryphracta deponiae*

10??00???0 ??????02?? ?0???1?4?? ??1102??20 ??0???0111 1?1??????? ?1?00??011 100?011010 0000101?01 ??00??0??0 0?00?000?0 ?1?1?000?? 1?1??0???? ???10???01 100001??1? ?0???????? 0?0?0?0??? ?1??11

*Stangerochampsa mccabei*

??00?1?21? 0101???21? ?000011010 001112??10 01000001?1 111??????? 11?0110011 1101021010 1000010121 11201?00?0 0200000000 0111?00010 1?1200?0?1 2?110???01 1000110?10 011??????? 000?001?12 ?11111

*Brachychampsa montana*

10001??211 11?10002?1 0?00011010 001?023110 10100?0111 111???00?1 11?00?0011 1101021010 1200010111 11201?0000 0200000100 0111000010 1012000001 12110?1?01 1000110?10 011??????? 000?001?12 ?11111

*Alligator mississippiensis*

1000110211 0101001201 1110111110 0011022130 1120100111 1111000011 1101000011 1101021010 2100000121 1110000010 0210010000 0111101010 1010000001 2211011001 1100010010 0210110011 0011010112 311111

*Navajosuchus mooki*

?????????? ??0????201 ?0???11?1? ??11021110 0100??01?1 111??????? ?1001??1?1 110?02100? 20000?0?21 11101?0000 0200?00?00 ?1???00??0 1?11???0?1 22110???01 1000110?10 0?1??????? 001?001??? ?11111

*Hassiacosuchus haupti*

00??1????1 ??????1201 1000?11?1? ??1??21?10 0?0???0111 111??????1 ?1?00??111 ????021000 2100?10??? ?1101???00 0200?0???? ?1????0??? 1?1????0?? 22?10?1?01 1000110?10 0????????? 0?1?001??? ??1?11

*Caiman yacare*

1000110211 1001011201 1101111110 0011021220 1122011111 0110100111 0100011011 1101021111 2300011121 1110100001 0211101000 0111010010 1010000001 2211011001 1000010010 2120111011 0011012112 311111

*Caiman latirostris*

1000110211 1000011201 110?111110 0011021220 1122011111 011010??1? ?100011011 1101021011 2300001121 1110100000 0211101000 0111010010 1010000001 2211011001 1010010010 2120121011 0011012112 311111

*Melanosuchus niger*

1000110211 1?01011201 1101111110 0011022220 1122011111 011010??11 1100011011 1101021011 2300001121 1110100000 0211101000 0111010010 1010100001 2211011001 1010010010 2120121011 0011012112 311111

*Paleosuchus trigonatus*

1000111210 0101111201 1001111312 1011021320 1220021111 0111110111 1100011011 1101021011 2200001121 1101111000 021?001100 0111010010 1010000001 2211011001 1000010010 2110112011 0011012112 311111

*Crocodylus cataphractus*

100001021? 001100?201 0100111212 0011021130 1011101001 011100100? 0100000001 1001100211 2010110110 0100200100 0210010010 0310000101 0110001111 0101011101 1000010110 0011010101 0101003112 311111

*Crocodylus porosus*

1110011211 0010001201 0100011212 0011021231 1011101001 0111001001 1100000001 1001100211 2010110110 0100100100 0210010010 0310000001 0110001111 0101011101 1001011110 0011010101 0101002112 311111

*Osteolaemus tetraspis*

??10011211 00100?1201 0100111112 0111021110 1011101001 0111001001 1100000001 1011100210 2010101110 0101011100 0210100001 0310000011 0110001111 0101011101 1001010011 0011010101 0111002112 311111

*Crocodylus robustus*

?????????? ??1????20? ??????111? ?111?????0 1011100011 011100??01 1100000001 1011100211 2110111110 01011?0100 0210010001 0310000001 0?10001111 0101011101 1001010011 001??????? 01110021?? 311111

*Crocodylus megarhinus*

????????0? ???????2?? ?????????? ?????????? 101???0001 011??????1 11?0000001 10002?0211 2?101?0100 01201?0100 0210000000 ?310000001 011000?11? 01010???01 1000010?10 001??????? 010?002??? 31?111

*Tomistoma schlegelii*

0210010211 0010001201 0000111111 0011021330 1040001001 0110000000 0100000201 1000010210 2010100010 0110200100 0210000001 0310000001 0110011111 0101011101 1000110010 0011010101 0101003112 311111

*Tomistoma lusitanica*

?????????? ???????2?? ?????????? ????02???0 ??4???0001 01???????? ?1?0000201 1000010211 20?1100010 01002?0100 0210000000 0310000101 011001?1?? 0101?11?01 1000110?10 001??????? 0?0?003??? 31?111

*Gavialosuchus americanus*

021001?210 001000020? ?01011?11? ??11?2???0 ??412????1 0111000??? ?1000002?1 100001021? 21?1110?10 0?102?0100 0210000000 031?0001?1 ??1000?1?? 0101????01 1000110?10 0011?????? 010?003112 31??11

*Crocodylus spenceri*

?????????? ???????2?? ?????????? ?????????? ??411?0?01 ??1??????1 ?1?0000??1 100???0210 20?0110010 01101?0?00 0?100??000 ?31??001?? ??100011?? ???10???01 1000110?10 001??????? 0?0?003??? 31?111

*Brachyuranochampsa eversolei*

?????????? ?????????? ?????????? ?????????? ?????????? ?????????? ?????????1 100?0?0210 2000100110 01001??000 02000?0?0? 031??00?0? 0?1?00?1?? ???10???01 1000110?10 001??????? 0?0?0????? ?1?111

*Crocodylus acer*

?????????? ?????????? ?????????? ?????????? ?????????? ?????????? ????????01 100?0?0211 2000?10110 01001?00?0 0?000?0001 031000000? 01100011?? 01010?1?01 1000110?10 001??????? 0?0?0????? ?1?111

*Crocodylus affinis*

0000010211 1011001201 0000011110 001102???0 1010000001 0111000001 1100000001 100?01011? 2000110110 01001?0000 0200000001 131?000000 011000?1?? 01010???01 1000010?10 0011?????? 010?002?12 311111

*Asiatosuchus germanicus*

001001?211 00????1201 ?000?11?1? ??11?????0 000???0001 011???00?? 11?00000?1 100?010000 1000?00?10 010?1?0000 0200000001 131??000?? 0?100??1?? 01?10???01 1000010?10 001??????? 000?000112 311111

*Prodiplocynodon langi*

?????????? ?????????? ?????????? ?????????? ?????????? ?????????? ?????????1 10??0?001? 10001?010? 01?01?00?? 02000?0001 131?00?01? 01100001?1 ???10?1001 1000?10?10 001??????? 0?0?0??1?? ?1?111

*Australosuchus clarkae*

?????1?2?? ???????2?? ?????1??1? ??1?02???0 10111?0001 011??????1 11?0000001 ????200211 2010?10?1? 01001?0?00 021000?000 0110?00001 011000?111 02010?1?01 1000110010 0?11?????? 010?002??2 ???111

*Aegyptosuchus peyerii*

?????????? ?????????? ?????????? ?????????? ?????????? ?????????? ?????????0 ?????????? 01?1?00??? ?10??????? ???0?????? ?????????? ??1??????0 2????????1 ??????0??? ?????????? ??1??????? ??????

*Aegisuchus witmerii*

?????????? ?????????? ?????????? ?????????? ?????????? ?????????? ?????????0 ???1?1???0 01?1?00??? ?10??????? ???0?????? ??1?????1? ?01???1?00 2????????1 ??????0??0 1????????? ??1??????? ???11?
